# Insights into the Chemistry of Iodine New Particle Formation: The Role of Iodine Oxides and the Source of Iodic Acid

**DOI:** 10.1021/jacs.1c12957

**Published:** 2022-05-23

**Authors:** Juan Carlos Gómez Martín, Thomas R. Lewis, Alexander D. James, Alfonso Saiz-Lopez, John M. C. Plane

**Affiliations:** † Instituto de Astrofísica de Andalucía, CSIC, Granada 18008, Spain; ‡ Department of Atmospheric Chemistry and Climate, Institute of Physical Chemistry Rocasolano, CSIC, Serrano 119, Madrid 28006, Spain; § School of Chemistry, University of Leeds, Leeds LS2 9JT, U.K.

## Abstract

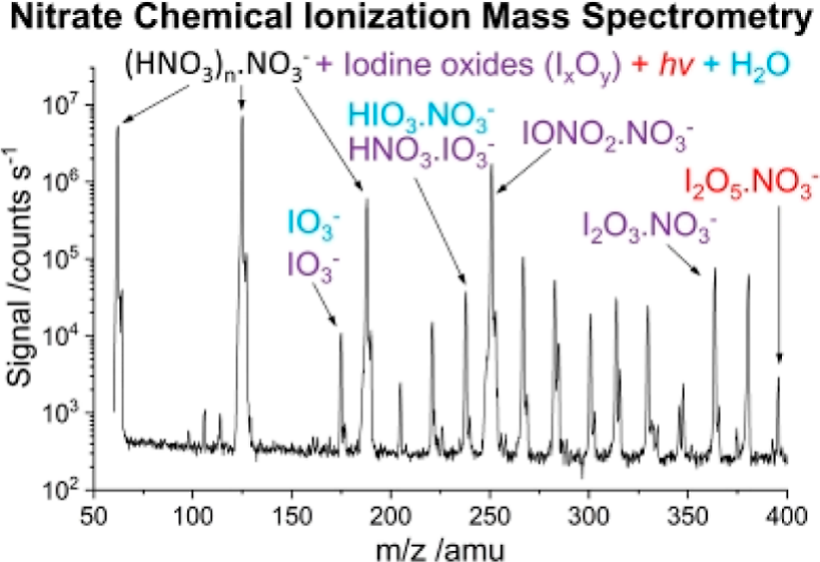

Iodine chemistry is an important driver of new particle formation in the marine and polar boundary layers. There are, however, conflicting views about how iodine gas-to-particle conversion proceeds. Laboratory studies indicate that the photooxidation of iodine produces iodine oxides (I_*x*_O_*y*_), which are well-known particle precursors. By contrast, nitrate anion chemical ionization mass spectrometry (CIMS) observations in field and environmental chamber studies have been interpreted as evidence of a dominant role of iodic acid (HIO_3_) in iodine-driven particle formation. Here, we report flow tube laboratory experiments that solve these discrepancies by showing that both I_*x*_O_*y*_ and HIO_3_ are involved in atmospheric new particle formation. I_2_O_*y*_ molecules (*y* = 2, 3, and 4) react with nitrate core ions to generate mass spectra similar to those obtained by CIMS, including the iodate anion. Iodine pentoxide (I_2_O_5_) produced by photolysis of higher-order I_*x*_O_*y*_ is hydrolyzed, likely by the water dimer, to yield HIO_3_, which also contributes to the iodate anion signal. We estimate that ∼50% of the iodate anion signals observed by nitrate CIMS under atmospheric water vapor concentrations originate from I_2_O_*y*_. Under such conditions, iodine-containing clusters and particles are formed by aggregation of I_2_O_*y*_ and HIO_3_, while under dry laboratory conditions, particle formation is driven exclusively by I_2_O_*y*_. An updated mechanism for iodine gas-to-particle conversion is provided. Furthermore, we propose that a key iodine reservoir species such as iodine nitrate, which we observe as a product of the reaction between iodine oxides and the nitrate anion, can also be detected by CIMS in the atmosphere.

## Introduction

Iodine gas-to-particle conversion is a fast process known since the early laboratory studies of iodine chemistry and spectroscopy.^1−3^ The nucleation rates of iodine oxide particles (IOPs) recently measured in the Cosmics Leaving Outdoor Droplets (CLOUD) chamber at the European Organization for Nuclear Research (CERN) suggest that this particle formation pathway can be competitive with sulfuric acid nucleation in pristine environments.^4^ In fact, atmospheric IOP particle formation unrelated to H_2_SO_4_ was observed for the first time in Mace Head (Ireland), a mid-latitude coastal location where tidal pool algae are exposed periodically to the atmosphere, resulting in strong biogenic emissions of iodine-bearing molecules that are photo-oxidized leading to low tide-day time particle “bursts”.^5,6^ Since then, there has been some debate about the potential climatic relevance of this phenomenon^7^ because iodine has been shown to be ubiquitous in the marine boundary layer (MBL).^8−10^ Although the atmospheric concentrations of gas-phase iodine species in the remote MBL are generally in the parts per trillion (ppt) range, new field observations in the Arctic demonstrate frequent new particle formation episodes triggered by iodine with little contribution from H_2_SO_4_.^11^ Hence, a regional influence of IOPs on cloud formation and properties over the polar oceans has been suggested, which could potentially accelerate sea ice melting.^4^ This could be exacerbated if the emissions of iodine from the ocean to the atmosphere are actually increasing, as indicated by Arctic and Alpine ice core measurements.^12,13^ Model efforts directed to evaluating the atmospheric radiative impact of IOPs are needed, but to do that, a feasible chemical mechanism connecting iodine emissions and gas-to-particle conversion is required.

Photolysis of iodine-bearing molecular precursors such as HOI, I_2_, CH_3_I, CH_2_I_2_, and so forth in the presence of ozone leads to the formation of iodine monoxide (IO), which has been observed in the MBL and in the polar regions,^7^ as well as in the free troposphere^14^ and lower stratosphere.^15^ Iodine dioxide (OIO) is a product of the IO self-reaction^16^ that has also been observed in the MBL.^17^ IO and OIO undergo rapid recombination reactions to generate higher-order iodine oxides (I_*x*_O_*y*_),^18^ which eventually form an ultrafine aerosol of I_2_O_5_ composition when formed in a dry environment.^19^ The composition of atmospheric IOPs is known to be iodic acid (HOIO_2_, hereafter HIO_3_ for simplicity), which is the hydrated form of I_2_O_5_.^20^ HIO_3_ has been detected in IOPs by photoionization mass spectrometry (PIMS).^21,22^

Recent chemical ionization mass spectrometry (CIMS) measurements confirm that IOPs consist almost entirely of HIO_3_ but have otherwise challenged the knowledge on gas-to-particle conversion summarized above.^4,23^ CIMS^24^ has revolutionized the detection of trace atmospheric constituents (e.g., H_2_SO_4_^25^) thanks to its extremely high sensitivity, soft ionization, and selective detection and has opened a new era beyond spectroscopic detection of atoms and simple molecules. The development of improved inlets, ionization sources, and atmospheric pressure interfaces has also enabled the detection of elusive gas-phase species, amongst which are iodine-containing molecules. CIMS field observations of the iodate anion (IO_3_^–^) have been interpreted by Sipilä et al.^23^ as a signature of HIO_3_ from an analogy with the detection of H_2_SO_4_ as HSO_4_^–^^25^ and based on ab initio proton affinities of NO_3_^–^ and IO_3_^–^
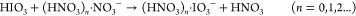
R1

The dominance of the IO_3_^–^ signal over that of other ions that can be linked to iodine oxides led Sipilä et al. to propose HIO_3_ as a major iodine-bearing molecule in the atmosphere. Reported HIO_3_ mixing ratios at Mace Head are comparable to or even higher than IO mixing ratios measured by laser-induced fluorescence.^26^ Sipilä et al. also reported the observation of the HIO_3_ dimer detected as HIO_3_·IO_3_^–^ and a mass peak progression that would be consistent with a nucleation mechanism where a cluster takes one HIO_3_ and upon addition of a second HIO_3_ sheds a water molecule. This mechanism has been amended recently considering the strong influence of instrumental settings on the observed mass spectra and currently also invokes iodous acid (HOIO, hereafter HIO_2_) to explain the observed mass peaks.^4^ Concurrent CIMS measurements with different ionization sources appear to support the existence of gas-phase HIO_2_ and HIO_3_ in the CLOUD experiments^4,27,28^ and by extension in the atmosphere.

There is however a major unknown about gas-phase HIO_3_: how does it form? The CIMS IO_3_^–^ signal has been observed in the absence of HO_*x*_ in laboratory flow tube experiments^23^ and in CLOUD,^4,28^ although the only known thermochemically feasible route from I_2_ photooxidation to iodic acid is the recombination reaction^29^
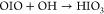
R2

It has then been postulated that HIO_3_ could be generated by a composite reaction involving I, O_3_, and H_2_O or by reactions between iodine oxides and water.^4,23^ However, atomic iodine and H_2_O form a very weakly bound complex that would not live long enough to react with atmospheric O_3_ (assuming no barriers in that reaction), and elementary reactions of iodine oxides with H_2_O generating HIO_3_ are endothermic or exhibit barriers, according to high-level electronic structure calculations.^22,30,31^ Hydrolysis of I_2_O_*y*_ by the water dimer has only been explored theoretically for I_2_O_5_,^32^ although to date it is unclear whether this species actually forms in the gas phase to play a role in IOP formation.^4,19,22,28^ Moreover, in our previous work, we were unable to detect gas-phase HIO_3_ by near-threshold PIMS at 11.6 eV, while we did detect it in the particle phase after pyrolysis of IOPs formed in a flow tube in the presence of water vapor.^22^ Gas-phase reactions between iodine species and H_2_O are, according to these experiments, slower than ∼10^–19^ cm^3^ molecule^–1^ s^–1^. In contrast, in our work, we demonstrated that iodine oxides (I_*x*_O_*y*_) readily form molecular clusters whose dry composition tends asymptotically to I_2_O_5_ (whose hydrated form is HIO_3_). We then proposed that the IOP formation mechanism that was commonly accepted before the CIMS observations still holds, i.e. IOPs are formed from I_*x*_O_*y*_, and the resulting I_2_O_5_ particles hydrate to form HIO_3_ in the particle phase.^22^ As a rebuttal to this conclusion, it has been argued that all laboratory studies on IOP formation have not been performed under atmospherically relevant conditions,^4^ implying that the iodine concentration in those studies was high enough for iodine oxides to drive IOP formation through dipole–dipole enhanced second-order chemistry. In principle, it is conceivable that under the low iodine and high water mixing ratios (ppt and %, respectively) typical of the lower atmosphere, a hypothetical reaction with a low rate constant between an iodine species and water vapor could proceed at a faster rate than the recombination of iodine oxides at ppt levels. There could even be a situation where both mechanisms could be competitive, and interestingly, CIMS also detects atmospheric I_*x*_O_*y*_ in the form of I_*x*_O_*y*_·NO_3_^–^ or I_*x*_O_*y*_·Br^–^, although these signals are uncalibrated.^4,28^

However, another possible explanation for the apparent contradiction between CIMS and PIMS gas-phase measurements is that the ions observed by CIMS may be generated, at least in part, by ion–molecule reactions between the reagent ion and iodine oxides. Our ab initio calculations indicated that different reactions between I_*x*_O_*y*_ with *x* = 2 and NO_3_^–^, Br^–^, CH_3_COO^–^, and H_3_O^+^ are exothermic and can potentially generate some of the ions and cluster ions that have been attributed to HIO_3_, in particular IO_3_^–^ and HIO_3_·NO_3_^–^ in the nitrate anion CIMS.^22,33^ For example,
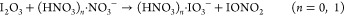
R3

If this was the case, the observations of these ions in the field using CIMS should be reinterpreted as being representative of both ambient I_2_O_*y*_ and HIO_3_. Moreover, this would call into question the need of invoking a gas-phase species of uncertain origin such as HIO_3_ to interpret signals that can be explained by other species whose formation is thermochemically unhindered. Hence, there is a clear need to carry out laboratory work on ion–molecule reactions that play a role in the different ionization schemes used by CIMS instruments.

Here, we present results from flow tube-mass spectrometry experiments performed to investigate the products of I_*x*_O_*y*_ ion–molecule reactions in the nitrate CIMS. Our results confirm our theoretical prediction that the IO_3_^–^ anion (*m*/*z* = 175) and the HIO_3_·NO_3_^–^ anion (*m*/*z* = 238), which we interpret as HNO_3_·IO_3_^–^, are generated from reactions between I_*x*_O_*y*_ and nitrate core ions. This implies that these ions cannot be exclusively attributed to ambient HIO_3_ and that the CIMS field observations need to be reinterpreted. We also identify the source of ambient HIO_3_. Finally, we observe a strong signal at *m*/*z* = 251, which corresponds to the ion cluster IONO_2_·NO_3_^–^,^34^ where iodine nitrate (IONO_2_) is formed in part as the coproduct of the iodate core anion in reaction R3. Hence, we propose that field CIMS instruments that have reported this signal^11^ may inadvertently have detected for the first time the key atmospheric iodine reservoir IONO_2_ (Saiz-Lopez et al., 2012), for which a detection technique has not been developed to date.

## Experimental Section

**Figure 1:**
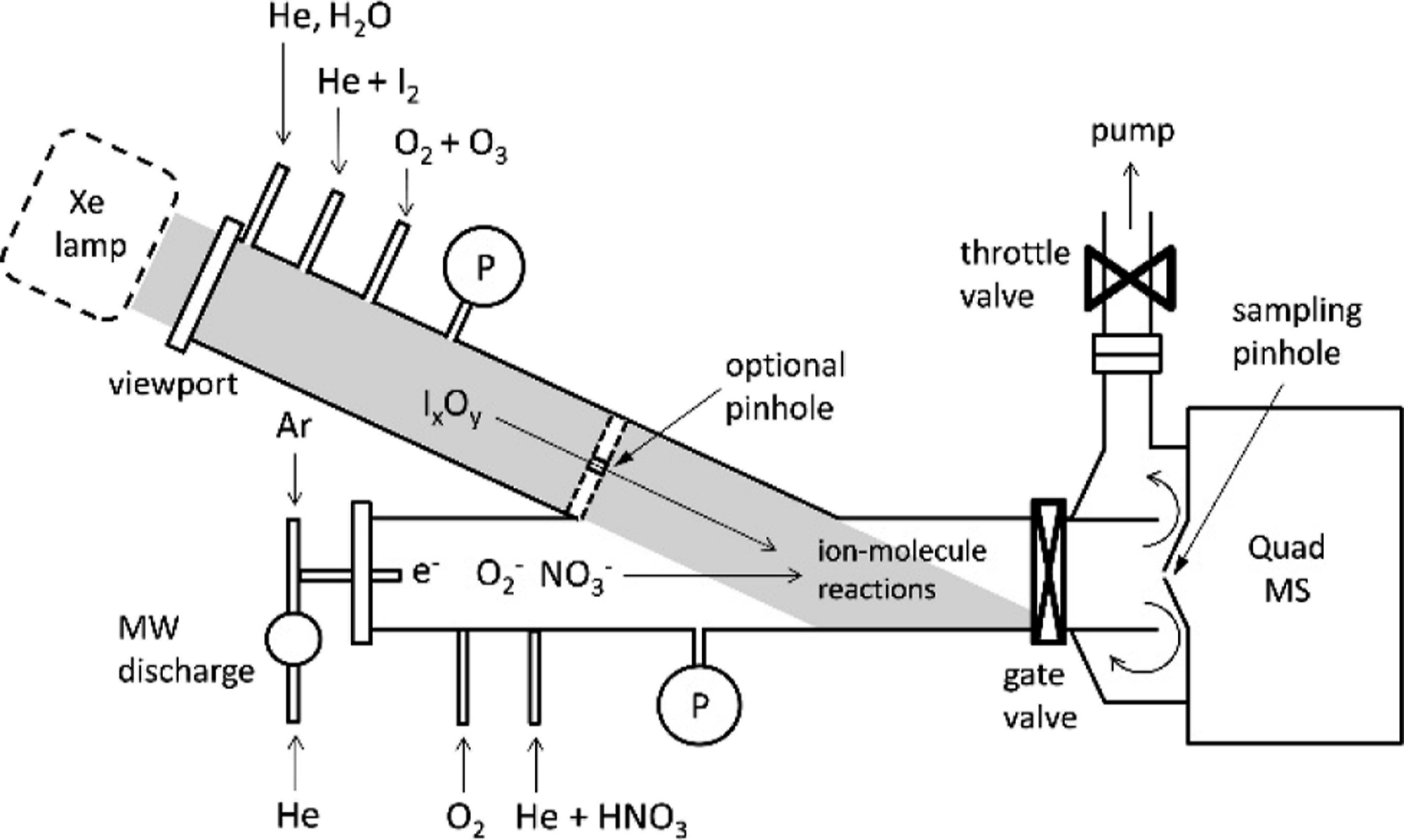
Flowing afterglow-fast flow tube experimental setup for ion–molecule reactions. The I_*x*_O_*y*_ branch could be operated at the same pressure as the NO_3_^–^ branch or at a higher pressure by inserting a pin-holed flange. P indicates pressure heads. Detection of negative ions was performed using a quadrupole mass spectrometer.

The interaction between NO_3_^–^ and I_*x*_O_*y*_ has been investigated by using the flowing afterglow technique, which we have used in the past to determine metal cation–electron recombination rate constants.^35,36^ Experiments are carried out in a Y-shaped 3.75 cm in diameter CF-flanged flow tube coupled to a quadrupole mass spectrometer (Hiden HPR 60). A schematic diagram of the apparatus is shown in Figure 1. A 200 W microwave (MW) discharge on He generates electrons (10^9^ to 10^10^ cm^–3^^35^), which are then carried by the He flow into the flow tube. A smaller flow of Ar (∼10% of the He flow) is added to quench excited He metastables generated in the MW plasma. The MW cavity is placed at 90° with respect to the flow tube to avoid irradiating the gas mixture with UV light emitted by the plasma. Once in the flow tube, the thermal electrons attach to O_2_ added through a side port, forming O_2_^–^, which further reacts with HNO_3_ added downstream of the O_2_ port to produce NO_3_^–^ and nitrate core ions^37^ with nearly 100% yield.^38^ The total flow through the NO_3_^–^ branch is typically 2–3 slm, and the pressure is kept around 3 Torr.

In the I_*x*_O_*y*_ branch of the flow tube, a flow of He (300–500 sccm) carrying I_2_ and O_3_ is continuously irradiated with white light from a 75 W Xe lamp (Photon Technology International) through a quartz view port. In a previous study, we used this setup to generate I_*x*_O_*y*_, which were detected by PIMS.^22^ An excess of I_2_ (10^12^ to 10^13^ molecule cm^–3^) removes on a ms time scale any OH generated by photolysis of O_3_ in the presence of residual or added water. The system can be operated in two pressure regimes. In the first one, I_*x*_O_*y*_ are generated at the same pressure as NO_3_^–^ (3 Torr) and the two flows are simply merged at the junction of the two branches. The residence time of the gas mixture in the I_*x*_O_*y*_ branch is 80–140 ms. In the second regime, a flange with a 1 mm pinhole is inserted upstream of the flow tube junction to raise the pressure up to 26 Torr, increasing the residence time to about 1.7 s. In both configurations, I_*x*_O_*y*_ (∼10^12^ cm^–3^) are generated well in excess of the concentration of NO_3_^–^ core ions (<10^7^ cm^–3^), and the pressure in the ion–molecule reaction region remains 3 Torr. The flows from the two branches are allowed to mix, and after a contact time of 12–21 ms, the gas is sampled through a skimmer cone with a 200 μm pinhole by the quadrupole mass spectrometer in a negative ion mode.

A roots blower (BOC Edwards, EH500A) backed with a rotary pump (BOC Edwards, E2M80) draws the gas down the flow tube. Flows are set using calibrated mass flow controllers (MKS), and the pressure is monitored using 10 and 1000 Torr calibrated capacitance manometers (MKS Baratron). The experiments are performed with CP grade He (BOC, 99.999%, [H_2_O] < 2 ppm) and N5 grade O_2_ (BOC, 99.999%, [H_2_O] < 1 ppm). Ozone is produced online by a corona discharge (EASELEC, ELO3G) of pure O_2_ at 1 bar. In some experiments, water vapor (deionized) is entrained in the flow tube by passing the carrier flow through a bubbler. Liquid HNO_3_ (Sigma-Aldrich, 99.5%) was stored in a glass finger container with 1/4″ connections in order to transfer it to a glass vacuum line equipped with 10 L glass bulbs. HNO_3_ is in equilibrium with NO_2_, which was removed by adding a few drops of H_2_SO_4_ (J.T. Baker, >51%). The glass finger was subsequently pumped for a few minutes before HNO_3_ vapor (vapor pressure of 30 Torr at 295 K) was released into the vacuum line in order to make up a diluted mixture in He (2%).

Data were acquired in the form of mass spectra in a negative ion mode usually in the range between 50 and 500 amu. Positive ion and neutral (electron impact ionization) mass spectra were also acquired for characterization of the flowing afterglow. Mass spectra were taken at 0.1 amu steps (10 accumulations). In some experiments, the signal of a set of selected peaks was followed in time to observe variations when changing the experimental conditions.

Electronic structure calculations were carried out to support the interpretation of the experimental data. The stationary points on the potential energy surfaces (PES) of selected reactions were first determined using the hybrid density functional/Hartree–Fock B3LYP method from within the Gaussian 16 suite of programs,^39^ combined with the standard 6-311+G(2d,p) triple-ζ basis set for O, N, and H, together with an all-electron basis set for I which was designed for G2 level calculations.^40^ This basis set may be described as a supplemented (15s12p6d)/[10s9p4d] 6-311G basis, the [5211111111,411111111,3111] contraction scheme being supplemented by diffused s and p functions, together with d and f polarization functions. Following geometry optimizations and determination of vibrational frequencies and (harmonic) zero-point energies, the energies of the stationary points relative to the reactants were obtained. Higher quality calculations of the relative energies of the reactants and products were made using the B3LYP functional and the significantly larger aug-cc-pVQZ basis set.^41^ For I, the aug-cc-pVQZ basis set of Peterson el al.^42^ was used. The accuracy of the reaction enthalpies calculated with this method is estimated here to be around ±20 kJ mol^–1^. A better accuracy may be expected for a large basis set such as aug-cc-pVQZ, but spin–orbit effects are not included, so this is likely a safe estimate. In a limited number of cases, fixed point CCSD(T) energy calculations have been carried out using the geometries optimized at the B3LYP/gen level (i.e., with the “G2” basis set).

## Results

### Dry Experiments

**Figure 2:**
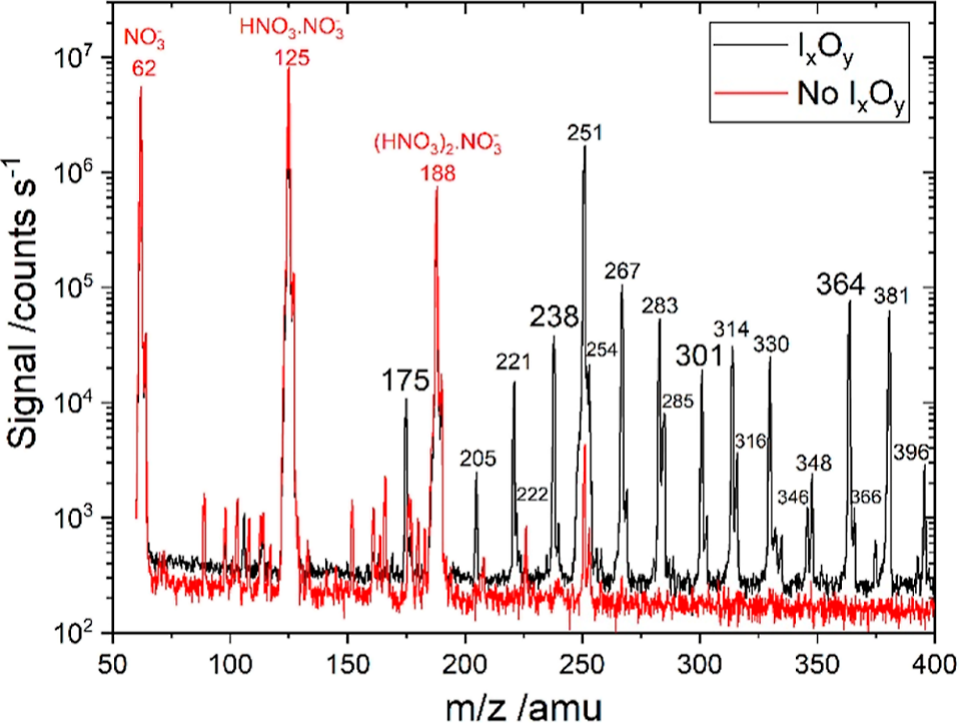
Mass spectrum of iodine oxide ions and iodine oxide-nitrate cluster ions (black line). Iodine oxides formed at 3 Torr after 137 ms and without addition of water vapor to the gas flow, prior to the ion–molecule reactions. Iodine-nitrate ions formed after 12 ms of the reaction time between the two gas flows. The spectrum of the nitrate core ion source (no I_*x*_O_*y*_) is also shown for comparison (red line). Note the logarithmic vertical scale.

Mass spectra recorded in the absence and presence of I_*x*_O_*y*_ without added water are shown in Figure 2. These experiments were run after pumping down the system to a few mTorr without having added any water prior to the observations. From mass spectrometric residual gas analysis (RGA) using electron impact ionization with and without adding water (e.g., Figure S1c), an upper limit to the water concentration in the I_*x*_O_*y*_ flow tube of 2 × 10^13^ molecule cm^–3^ is estimated (i.e., 4 orders of magnitude lower than atmospheric concentrations).

Table 1 lists the mass peaks shown in Figure 2 (“dry”) with the corresponding ion assignment and the proposed parent molecule. In these experiments, the pressure in the I_*x*_O_*y*_ branch was the same as in the NO_3_^–^ branch (3 Torr). In the absence of iodine oxides, the spectra show the expected peak progression of nitrate core ion peaks at *m*/*z* = 62 (NO_3_^–^), *m*/*z* = 125 (HNO_3_·NO_3_^–^), *m*/*z* = 188 ((HNO_3_)_2_·NO_3_^–^), and *m*/*z* = 251 ((HNO_3_)_3_·NO_3_^–^). The relative signal at *m*/*z* = 62 and *m*/*z* = 125 peak is determined by pressure and residence time of the gas in the flow tube, with higher pressure and slower flow promoting (HNO_3_)_*n*_·NO_3_^–^ (Figure S2a,b).

**Table 1:** Observed Peaks and Intensities, Dependence on Light and Humidity, and Assigned Parent Molecules

this work								CIMS literature			
peak^a^	anion	*m*/*z*	Int^b^	no O_3_^c^	dark	dry	H_2_O^d^	parent^e^	FT^f^	EC^g^	F^h^
127	I^–^	126.9	5–6	yes	yes	yes		I_2_			
143	IO^–^	142.9	2–3	no	yes	yes		I_2_	yes		
145	H_2_O·I^–^	144.9	2–3	no	yes	no		I_2_			
163	(H_2_O)_2_·I^–^	162.9	2–3	no	yes	yes		I_2_			
175	IO_3_^–^	174.9	4–5	no	yes	yes	↑	I_2_O_*y*; *y*=2–5_; HIO_3_	yes	yes	yes
190	HNO_3_·I^–^	189.9	3–4	yes	yes	yes	↑	I_2_			
205	IO·NO_3_^–^	204.9	3–4	yes	yes	yes	*	IO		yes	
221	OIO·NO_3_^–^	220.9	4–5	yes	yes	yes	*	OIO	p	yes	yes
222	HNO_3_·IO_2_^–^; HIO_2_·NO_3_^–^	221.9	3–4	yes	yes	yes	*	I_2_O_2_; HIO_2_		yes	yes
238	HNO_3_·IO_3_^–^; HIO_3_·NO_3_^–^	237.9	4–5	no	yes	yes	↑	I_2_O_3_; HIO_3_	p	yes	yes
251	IONO_2_·NO_3_^–^i	250.9	5–6	yes	yes	yes	*	I_2_O_3_	p	yes	yes
254	I_2_^–^	253.8	3–4	yes	yes	yes	↓	I_2_			
267	OIONO_2_·NO_3_^–^	266.9	4–5	yes	yes	yes	*	I_2_O_4_	p	yes	yes
283	O_2_IONO_2_·NO_3_^–^	282.9	3–4	no	yes	yes	↓	I_2_O_5_	p	no	yes
285	(HNO_3_)_2_·IO_2_^–^; HIO_2_·(HNO_3_)·NO_3_^–^	284.9	3–4	yes	yes	yes	↑	I_2_O_2_; HIO_2_	p	yes	yes
301	(HNO_3_)_2_·IO_3_^–^; HIO_3_·(HNO_3_)·NO_3_^–^	300.9	3–4	no	yes	yes	↑	I_2_O_*y*; *y*=2,3_; HIO_3_	p	yes	yes
314	IONO_2_·HNO_3_·NO_3_^–^	313.9	3–4	yes	yes	yes	*	I_2_O_3_	p		
316	I_2_·NO_3_^–^	315.8	2–3	yes	yes	yes	↔	I_2_			
330	OIONO_2_·HNO_3_·NO_3_^–^	329.9	3–4	yes	yes	yes	*	I_2_O_4_	p		
334	IO_2_·IO_3_^–^	333.8	2–3	no	no	no	↑	HIO_3_·OIO	p		
346	O_2_IONO_2_·HNO_3_·NO_3_^–^	345.9	2–3	yes	yes	yes	*	I_2_O_5_			
348	I_2_O_2_·NO_3_^–^	347.8	3–4	no	yes	yes	↔	I_2_O_2_		yes	
351	HIO_3_·IO_3_^–^	350.8	1–2	no	no	no	↑	(HIO_3_)_2_	yes		
364	I_2_O_3_·NO_3_^–^	363.8	3–4	no	yes	yes	↔	I_2_O_3_	p	yes	
366	I_2_O_2_·H_2_O·NO_3_^–^	365.8	1–2	no	no	no	↑	I_2_O_2_·H_2_O			
380	I_2_O_4_·NO_3_^–^j	379.8	2–3	no				I_2_O_4_	p	yes	yes
381	I_3_^–^	380.7	5–6	yes	yes	yes	*	I_2_			
396	I_2_O_5_·NO_3_^–^	395.8	3–4	no	no	yes	↓	I_2_O_5_	yes	yes	yes
398	I_2_O_4_·H_2_O·NO_3_^–^; H_2_I_2_O_5_·NO_3_^–^	397.8	2–3	no	yes	no	↑	I_2_O_4_·H_2_O; H_2_I_2_O_5_	p	yes	yes
411	I_2_O_2_·HNO_3_·NO_3_^–^	410.8	2–3	no	yes	yes	↔	I_2_O_2_	p		
427	I_2_O_3_·HNO_3_·NO_3_^–^	426.8	2–3	no	yes	yes	↔	I_2_O_3_	p	yes	
440	(IONO_2_)_2_·NO_3_^–^;IONO_2_·(HNO_3_)_3_·NO_3_^–^	439.8	2–3	no	yes	yes	*	I_2_O_3_			
442	OIO·O_2_IONO_2_·NO_3_^–^	441.8	2–3	no	no	yes	↓	OIO; I_2_O_5_			
443	I_2_O_4_·HNO_3_·NO_3_^–^	442.75	2–3	yes	yes	yes	↔	I_2_O_4_	p	yes	yes
456	OIONO_2_·(HNO_3_)_3_·NO_3_^–^	455.9	2–3	no	yes	yes	↔	I_2_O_4_			
461	H_2_I_2_O_5_·HNO_3_·NO_3_^–^; I_3_O_5_^–^	460.75	2–3	no	no	yes	↓	H_2_I_2_O_5_; HIO_3_·I_2_O_2_	p	yes	
477	(HIO_3_)_2_·HNO_3_·NO_3_^–^; I_3_O_6_^–^	476.75	1–2	no	no	yes	*	HIO_3_; HIO_3_·I_2_O_3_	p		
488	OIONO_2_·O_2_IONO_2_·NO_3_^–^	487.8	2–3	no	no	yes	↓	I_2_O_4_ and I_2_O_5_			
493	I_3_O_7_^–^	493.7	1–2	no	no	no	↑	HIO_3_·I_2_O_4_	p		

a Integer mass (number of neutrons + number of protons).

b Average peak intensity logarithmic range (*x*–*y* indicates the signal between 10^*x*^ and 10^*y*^).

c Indicate if the anion signal is above the detection limit without O_3_ in the dark and without adding H_2_O.

d Indicates the effect of adding H_2_O on the photolytic signal of each anion after correcting for the effect of H_2_O on the nitrate core ions: increase (↑), decrease (↓), no change (↔), and unclear (*).

e Refers to neutral molecules from the I_*x*_O_*y*_ flow tube that originate in the observed ion.

f Flow tube CIMS: Sipilä et al. 2016 (Figure S4). “Yes” indicates positive detection. Since no table is provided in the original paper, the figure has been digitized; “p” indicates possible detection (i.e., there is a mass in the mass defect plot very close to the mass in the first column of the present table).

g Environmental Chamber CIMS: He et al. 2021, Table S2 and Figure S4.

h Field CIMS: Baccarini et al. 2020, Table S1.

i Overlaps with (HNO_3_)_3_·NO_3_^–^.

j Overlaps with I_3_^–^, but it can be observed by subtraction of mass spectra.

Addition of molecular iodine to the flow results in a substantial decrease in the nitrate core ion peaks (Figure S3a) and concurrent appearance of new mass peaks. Peaks at *m*/*z* = 127, 254, and 381 indicate the presence of I^–^, I_2_^–^, and I_3_^–^, respectively. The latter is a prominent signal that has also been observed in iodine-based CIMS.^34^ The peak at *m*/*z* = 251 increases by 2 orders of magnitude, and we identify it now as the halogen-bonded complex IONO_2_·NO_3_^–^ observed in previous CIMS work when I_2_ and NO_3_^–^ are present in sampled air.^34^ Other minor masses observed are *m*/*z* = 205 (IO·NO_3_), *m*/*z* = 221 (OIO·NO_3_^–^), *m*/*z* = 222 (HNO_3_·IO_2_^–^), *m*/*z* = 254 (I_2_^–^), *m*/*z* = 267 (OIONO_2_·NO_3_^–^), *m*/*z* = 314 (IONO_2_·HNO_3_·NO_3_^–^), *m*/*z* = 316 (I_2_·NO_3_^–^), *m*/*z* = 440 ((IONO_2_)_2_·NO_3_^–^ or IONO_2_·(HNO_3_)_3_·NO_3_^–^), and *m*/*z* = 443 (I_2_O_4_·HNO_3_·NO_3_^–^). The oxidation of I_2_ is not photochemical but caused by surface chemistry following I_2_ deposition on the wall downstream of the ionization region (note that the gas-phase reaction NO_3_^–^ + I_2_ → IONO_2_ + I^–^ is endothermic using evaluated enthalpies of formation^43,44^).

When iodine oxides are made by adding ozone to the flow, additional peaks of iodine-containing ions emerge, and most peaks that had appeared in the presence of I_2_ (Figure S3a) increase substantially (Figure S3b). Irradiation with the Xe lamp beam enhances the signals by a factor of 1.5–2.5 (Figure S4a,c), except for I_3_^–^, which decreases by ∼5%. This means that I_*x*_O_*y*_ are generated in this system both by a dark reaction between I_2_ and O_3_ and by gas-phase photochemistry^22^ within a residence time of tens to hundreds of milliseconds. The gas-phase reaction between I_2_ and O_3_ is slow,^18,45^ which means that additional wall chemistry is taking place in this system. The flow is not turbulent (Reynold numbers are low), but radial diffusion is favored by relatively low pressures and by the use of He as a carrier gas. This dark source of I_*x*_O_*y*_ helps to pinpoint species generated exclusively by photochemistry.

The new masses that appear in the mass spectra when I_*x*_O_*y*_ are made by ozone and/or irradiation are *m*/*z* = 175 (IO_3_^–^), *m*/*z* = 238 (HNO_3_·IO_3_^–^), *m*/*z* = 301 ((HNO_3_)_2_·IO_3_^–^), *m*/*z* = 283 (O_2_IONO_2_·NO_3_^–^), *m*/*z* = 348 (I_2_O_2_·NO_3_^–^), *m*/*z* = 364 ((HNO_3_)_3_·IO_3_^–^ and I_2_O_3_·NO_3_^–^), *m*/*z* = 396 (I_2_O_5_·NO_3_^–^), *m*/*z* = 411 (I_2_O_2_·HNO_3_·NO_3_^–^), and *m*/*z* = 427 (I_2_O_3_·HNO_3_·NO_3_^–^) (Figure S3b). Of the three iodate core ion peaks, the most prominent one is generally HNO_3_·IO_3_^–^. The I_2_O_5_·NO_3_^–^ anion is only generated in the presence of light (Figure S4a,c). Other minor peaks are detected at higher *m*/*z* (see Figure S4b,d and Table 1).

Decreasing the ozone or the iodine concentrations results in the reduction of all these ions and also of IONO_2_·NO_3_^–^, which shows the same behavior as the (HNO_3_)_*n*_·IO_3_^–^ ions on top of its background signal (see time traces in Figure S5). By contrast, I_3_^–^ increases with lower ozone and with a higher I_2_ concentration and can be used as a proxy for I_2_. Reducing the reaction time by injecting the ozone flow further downstream results in reduction of most signals (Figure S4) both for the dark and the photolytic source. It should be noted that because I_2_, O_3_, and I_*x*_O_*y*_ are in excess over the available charged species, variations of the conditions in the I_*x*_O_*y*_ flow tube may also change the available charge and the relative concentrations of the nitrate core ions. For example, adding more I_2_ may reduce the (HNO_3_)_*n*_·NO_3_^–^ ions available for reaction with I_*x*_O_*y*_ (Figure S3a shows that the (HNO_3_)_*n*_·NO_3_^–^ signals decrease when I_2_ is added). Also, a higher pressure or a slower flow in the ion source flow tube promotes the (HNO_3_)_*n*_·NO_3_^–^ ions versus NO_3_^–^, and in the ion–molecule reaction region, clustering of ions and molecules is favored over dissociation. A longer residence time may, on the other hand, enhance reactive and diffusive loss of ions. When the two branches of the experiment are at the same pressure, all these effects overlap in the observed mass spectra. Thus, the observed changes in the (HNO_3_)_*n*_·IO_3_^–^ or I_*x*_O_*y*_·NO_3_^–^ signals may not only result from varying I_*x*_O_*y*_ but also from varying (HNO_3_)_*n*_·NO_3_^–^. This is illustrated in Figure S2, which shows mass spectra for two experiments where I_*x*_O_*y*_ form under the same conditions but the flow through the ion source differs by a factor of two. A slower flow enhances the signals of the heavier ions, reduces the signals of the (HNO_3_)_*n*_·IO_3_^–^ ions, and also changes the signal ratios between the latter.

**Figure 3:**
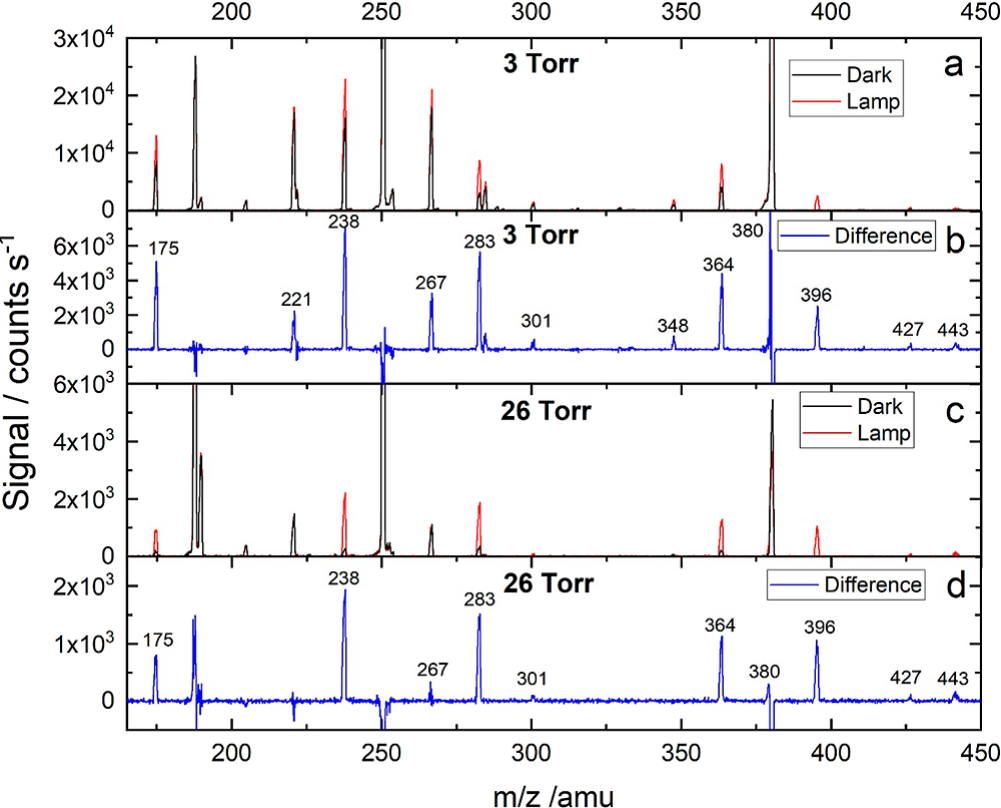
Mass spectra of iodine oxide ions, where iodine oxides were generated at 3 Torr (a,b) or at 26 Torr (c,d). Panels a and c show the raw spectra obtained in the dark (black lines) and by irradiating the tube axially with white light (red lines). Panels b and d show the photolytic signal, that is, the difference between the signals recorded with and without light.

Keeping the I_*x*_O_*y*_ branch of the flow tube behind a pin-holed wall (Figure 1) has several advantages, which include the ability of changing pressure in the I_*x*_O_*y*_ formation region without affecting pressure in the ion source and avoiding illumination of the ion–molecule reaction volume. Moreover, in the higher-pressure experiments (26 Torr), I_*x*_O_*y*_ were mostly generated by gas-phase photochemistry (e.g., a five to ten times more photolytic HNO_3_·IO_3_^–^ signal than from the dark reaction, compare Figure 3a,c) owing to enhanced I_2_ photolysis (∼30%) resulting from the longer residence time (1.7 s) and to reduced wall interaction as a result of slower molecular diffusion at a higher pressure. In the 26 Torr experiments, the flows through the iodine trap and the ozone generator were reduced to maintain a similar concentration of I_*x*_O_*y*_ as in the 3 Torr experiments to avoid build-up of particles that could block the pinhole.^18^ The ion–molecule reaction products in both experiments are the same (same peaks in Figures 3a,c), but the signals of the iodine-containing anions are smaller relative to the nitrate core ion signals in the 26 Torr experiments (signals are shown normalized to the NO_3_^–^ signal in Figure 3) for similar contact time in the ion–molecule reaction region, suggesting a different distribution of products in the I_*x*_O_*y*_ flow tube. Regarding the photolytic signals in the higher-pressure experiments, 30% photolysis of I_2_ results in 7% less background IONO_2_·NO_3_^–^ in the experiments with light and hence the negative peak in the difference spectrum at *m*/*z* = 251 (Figure 3d).

### Wet Experiments

Similar to our results above, the first observation in a laboratory setting of IO_3_^–^ by nitrate CIMS analysis of an I_2_ + O_3_ mixture took place without actively adding water to the flow tube.^23^ Interpretation of IO_3_^–^ as HIO_3_ requires a source of hydrogen atoms. Hence, in the absence of HO_*x*_, the formation of HIO_3_ was explained by Sipilä et al.^23^ as the result of a very fast reaction between I_2_, O_3_, and water degassed from the walls of the flow tube ([H_2_O] < 8 × 10^15^ molecule cm^–3^). Subsequent experiments were conducted where increasing water vapor concentrations up to 4 × 10^16^ molecule cm^–3^ were added to the flow tube. This resulted in a factor of two increase of the raw (not charge-normalized) IO_3_^–^ signal, which was seen as a confirmation of the need of water to form HIO_3_.^23^

In order to investigate the effect of water in our system, the I_*x*_O_*y*_ carrier gas was humidified by passing it through a bubbler containing deionized water, at the same pressure as the flow tube (i.e. the bubbler is downstream of the carrier gas flow controller). The water vapor concentration in the I_*x*_O_*y*_ branch at 3 Torr is estimated from the pressure variation to be ∼8 × 10^15^ molecule cm^–3^. The minimum water concentration in these experiments, where water was turned on and off several times, is estimated from the ratios of the H_2_O·NO_3_^–^ ion cluster signal, and found to be 1 order of magnitude higher than in the “dry” experiments. The estimated concentration of water vapor at 26 Torr is ∼2.5 × 10^17^ molecule cm^–3^, corresponding to the atmospheric water vapor concentration for RH = 33% at 760 Torr and 25 °C. Addition of water to the ion–molecule reaction volume ([H_2_O] ∼ 1 × 10^15^ molecule cm^–3^ after dilution by the larger flow that passes through the ion source) results in a general increase of the nitrate core ion signals, as shown in Figure S1. The NO_3_^–^ and HNO_3_·NO_3_^–^ signals increase by a factor of ∼2. A possible explanation of this observation is that water slows down anion–cation neutralization by forming clusters with negative and positive ions (Figure S1a,b). Another possibility is that water deposition passivates the inner surfaces in the ion–molecule reaction volume, reducing the wall loss of anions.

**Figure 4:**
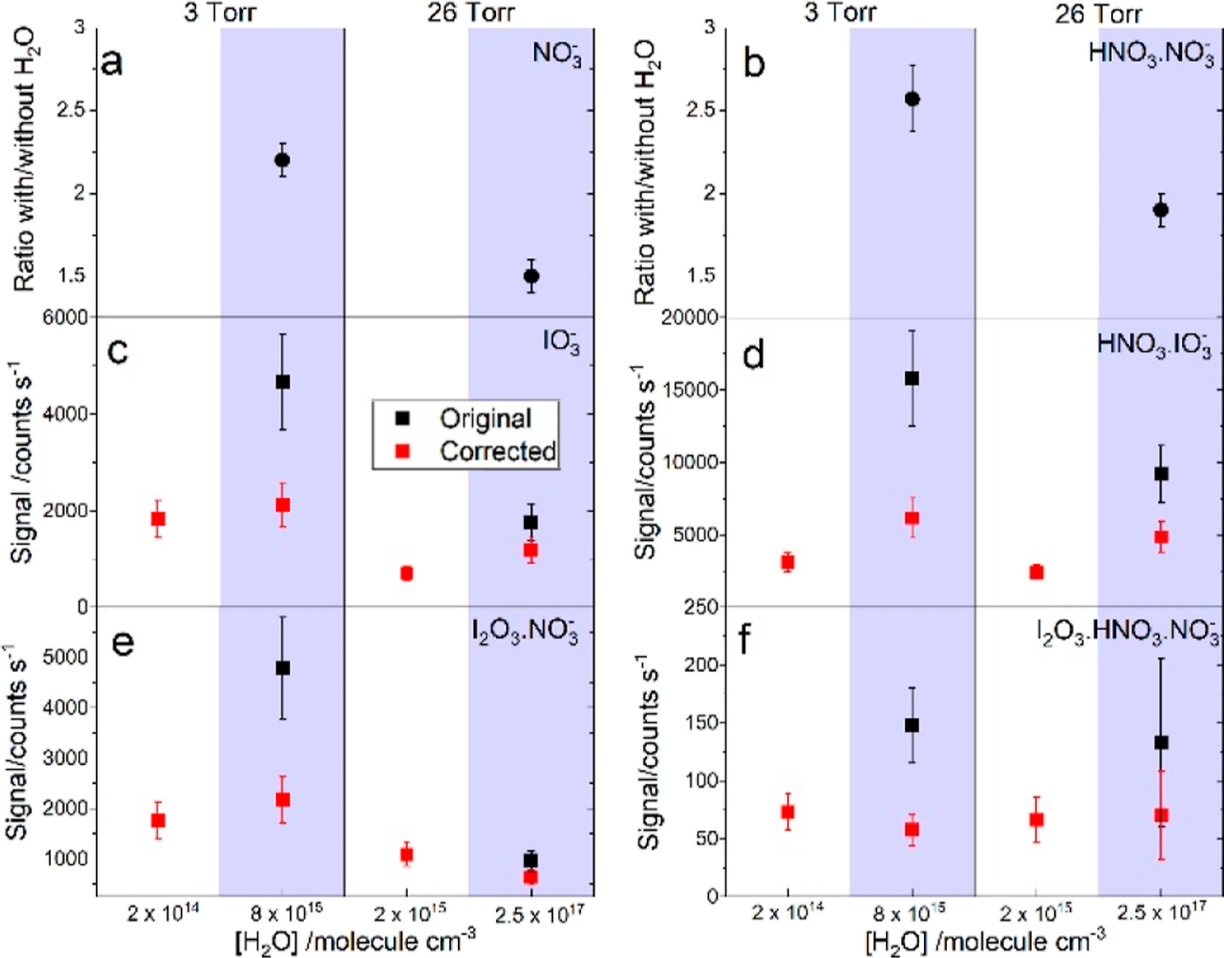
Water dependence of nitrate core anions and selected iodine oxide anions for two experiments at 3 and 26 Torr. Panels a and b show, respectively, the ratios between the NO_3_^–^ and HNO_3_·NO_3_^–^ signals (i.e., the integrated area under a mass peak) measured with (shaded blue) and without water. Panels c and d show the IO_3_^–^ and HNO_3_·IO_3_^–^ photolytic signals obtained from the raw spectra (black squares) and corrected with the nitrate core ion ratios in panels a and b, respectively. Panels e and f show the same as panels c and d for I_2_O_3_·NO_3_^–^ and I_2_O_3_·HNO_3_·NO_3_^–^.

Mass spectra obtained with and without water at 3 and 26 Torr are shown in Figure S6. The contribution of the dark reaction has been removed from these spectra, and only photolytic signals are shown. Addition of water enhances the iodate core ion signals by a factor of ∼3 in both experiments, while the I_2_O_5_·NO_3_^–^ and O_2_IONO_2_·NO_3_^–^ signals reduce upon addition of water. Figure 4 shows that scaling the IO_3_^–^ and HNO_3_·IO_3_^–^ signals with measured NO_3_^–^ and HNO_3_·NO_3_^–^ enhancement factors in the presence of water (equivalent to the usual normalization to the available charge performed in CIMS measurements) significantly reduces the difference between the dry and wet observations. After correction, the iodate core ion signals in the presence of water are still up to two times higher, both in the 3 Torr and the 26 Torr experiments. This may be an indication of formation of HIO_3_ followed by RR1.

**Figure 5:**
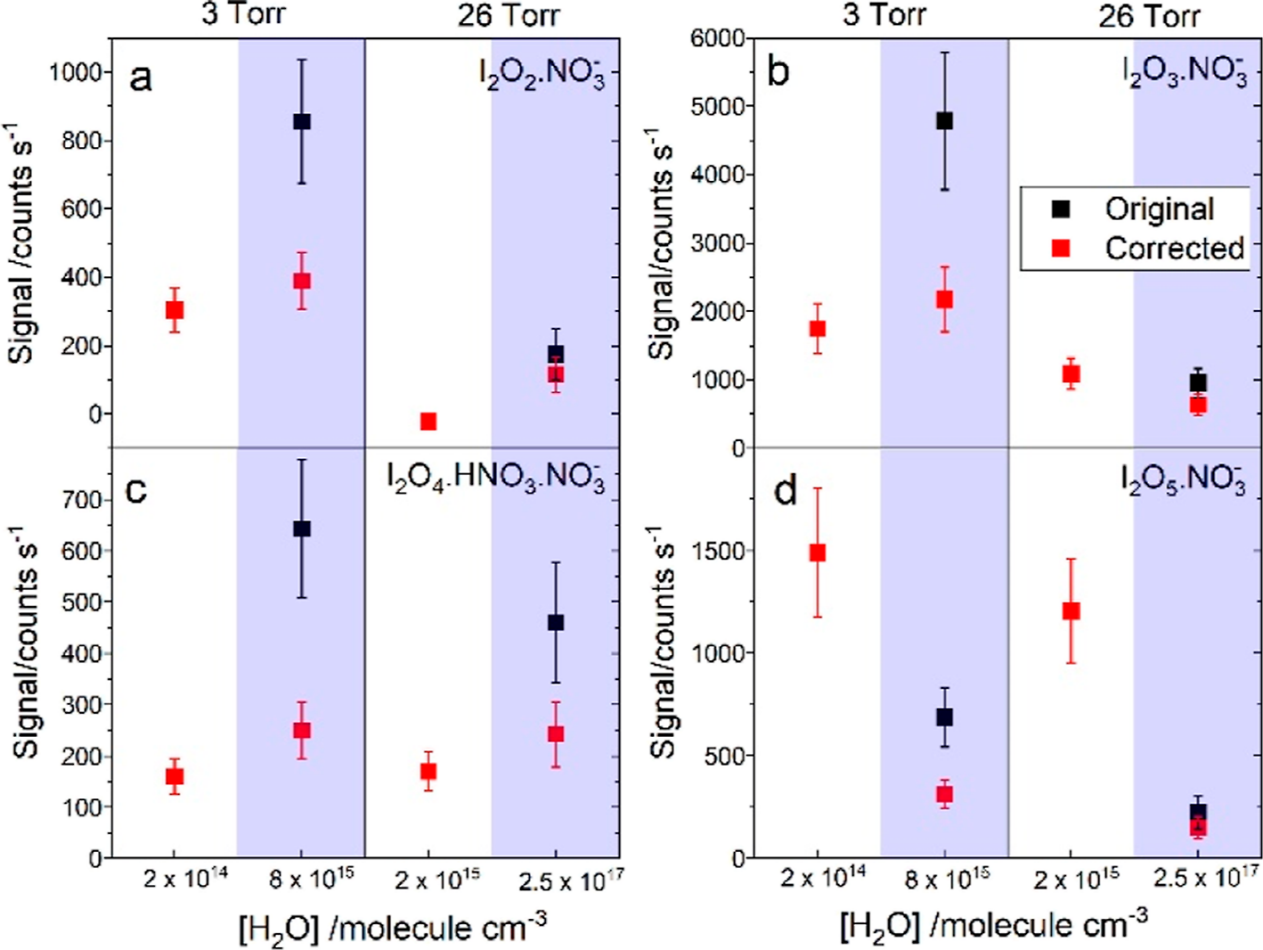
Water dependence of I_*x*_O_*y*_·(HNO_3_)_*n*_·NO_3_^–^ photolytic signals for two experiments at 3 and 26 Torr: I_2_O_2_·NO_3_^–^ (panel a), I_2_O_3_·NO_3_^–^ (panel b), I_2_O_4_·HNO_3_·NO_3_^–^ (panel c), and I_2_O_5_·NO_3_^–^ (panel d). Black squares: signals obtained by integrating the corresponding mass peaks. Red squares: signals corrected with the NO_3_^–^ ratios with/without water shown in Figure 4a,b.

To complete this picture, we include in Figure 4 the corresponding I_2_O_3_·NO_3_^–^ and I_2_O_3_·HNO_3_·NO_3_^–^ measurements, which after correction show no difference with the values under dry conditions. Similarly, the I_2_O_2_·NO_3_^–^, and I_2_O_4_·HNO_3_·NO_3_^–^ measurements in the presence of water remain close to the dry values after applying the corresponding scaling factor (Figure 5). This means that water does not remove I_*x*_O_*y*_ (*y* = 2–4). The only I_2_O_*y*_–related signal that is significantly reduced by water systematically is that of the I_2_O_5_·NO_3_^–^ anion (Figure 5d), whose parent neutral is I_2_O_5_. The decrease of the I_2_O_5_·NO_3_^–^ signal and the increase of the IO_3_^–^ signal upon addition of water suggest that the loss of I_2_O_5_ results in the formation of HIO_3_. This is supported by the lack of increase of the iodate core ion signals in the absence of light (Figure S7a,c), where I_2_O_5_ does not form (Figure S7f), but other I_2_O_*y*_ do.

There are other important observations in our experiments regarding the molecular clusters that have been proposed as the initial steps in the oxyacid-driven IOP nucleation mechanism. With light and in the presence of water, we observe a small peak at *m*/*z* = 351 that could be attributed to the HIO_3_ dimer.^4,23^ There are also other peaks that appear with light and added water that may be related to clusters formed by addition of HIO_3_ to iodine oxides (*m*/*z* = 334, *m*/*z* = 477, and *m*/*z* = 494, see Table 1). In particular, the peak at *m*/*z* = 398 (HIO_2_·HIO_3_·NO_3_^–^ or I_2_O_4_·H_2_O·NO_3_^–^) only appears in the presence of water.

## Discussion

### Interpretation of Mass Spectra Obtained without Added Water Vapor

Some of the masses listed in Table 1 (*m*/*z* = 205, 221, 348, 364, and 380) result from clustering between well-known iodine oxides^18,46,47^ and nitrate ions in the ion–molecule reaction volume and have been reported in previous CIMS studies^4,11^
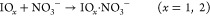
R4
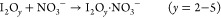
R5

The observation of I_2_O_5_ in the form of I_2_O_5_·NO_3_^–^ is somewhat surprising since gas-phase I_2_O_5_ was not unambiguously observed by PIMS under similar conditions.^18,22^ This mass is observed both at 3 and 26 Torr only if the mixture is irradiated (Figure S7) and is not formed from the dark I_2_ + O_3_ reaction as is the case for the other I_2_O_*y*_, which indicates that I_2_O_5_ is a gas-phase photolysis product of a higher-order iodine oxide such as I_3_O_7_.^48^ We note that I_3_O_*n*_ (*n* = 5–7) have been previously observed both by PIMS as I_3_O_*n*_^+^^18,22^ and by nitrate CIMS as I_3_O_*n*_·NO_3_^–^ (*m*/*z* > 500 amu).^23^

Three prominent iodine-containing ions are IO_3_^–^ (*m*/*z* = 175), HNO_3_·IO_3_^–^ (*m*/*z* = 238), and (HNO_3_)_2_·IO_3_^–^ (*m*/*z* = 301). These masses have been previously observed with nitrate CIMS instruments^4,23^ and have been interpreted as products of ion–molecule reactions between HIO_3_ and (HNO_3_)_*n*_·NO_3_^–^ (*n* = 0–2) reaction R1 in the instrument inlet. Any OH generated by UV photolysis of O_3_ in the presence of water in our experiments is scavenged by I_2_ and therefore cannot generate HIO_3_ via reaction R2. This leaves water as the only other possible reagent. For water concentrations as low as those in the “dry” experiments at 3 Torr ([H_2_O] < 2 × 10^13^ cm^3^) and a reaction time of 130 ms in the I_*x*_O_*y*_ flow tube, the rate constant of any hypothetical gas-phase mechanism forming HIO_3_ from water plus I (+O_3_), IO, OIO, or I_2_O_*y*_ (*y* = 2–4) where the reaction with water is rate limiting would have an effective rate constant of *k* ≥ 4 × 10^–13^ cm^3^ molecule^–1^ s^–1^. This is clearly at odds with the upper limits to the effective rate constants of reactions between atomic iodine (+O_3_) or iodine oxides and water, forming HIO_3_, which were found to be lower than ∼10^–19^ cm^3^ molecule^–1^ s^–1^.^22^ HIO_3_ could also be formed by hydrolysis of I_*x*_O_*y*_ on the surfaces of the flow tube, although no HIO_3_ from the gas phase or surface chemistry was observed by PIMS in the same system. Furthermore, Born–Oppenheimer molecular dynamics simulations indicate that I_2_O_*y*_ reactions at the air–water interface do not take place.^22^ Therefore, it is likely that masses 175, 238, and 301 result from ion–molecule reactions between iodine oxides, which are detected in our system both by PIMS and CIMS, and nitrate core ions:
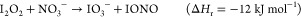
R6.1
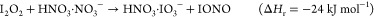
R6.2
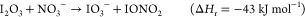
R3.1
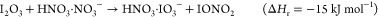
R3.2
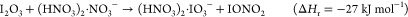
R3.3
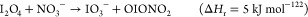
R7

**Figure 6:**
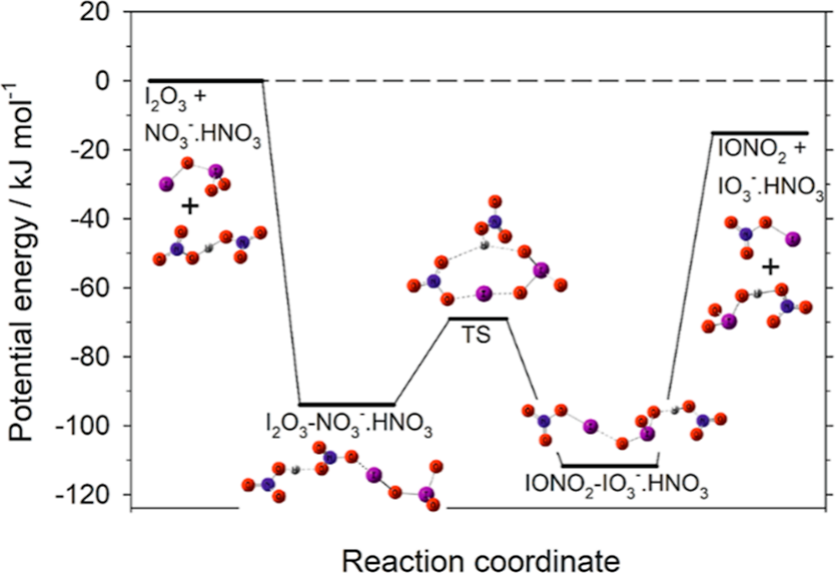
Potential energy for reaction R3.2 at the B3LYP/aug-cc-pVQZ level of theory (see Table S3 for further details).

Ab initio enthalpies of reactions I_2_O_*y*_ + NO_3_^–^ were reported in our previous publication (Supporting Information of Gómez Martín et al.,^22^). These were calculated at B3LYP/6-311+G(2d,p) level of theory with the iodine basis set mentioned above^40^ and validated with evaluated thermochemical data. Higher level of theory calculations (CCSD(T)/aug-cc-pVTZ + LANL2DZ//M06-2X/aug-cc-pVDZ + LANL2DZ) confirmed that the reaction R3.1, facilitated by the formation of a IO_3_–IONO_2_ halogen-bonded adduct, is exothermic and barrierless.^33^ This is not too surprising, considering that halogen bonding has been found to play an important role in the iodine CIMS when used for detecting HNO_3_.^34^ Here, we have revisited our previous calculations^22^ and extended them to reactions R6.2, R3.2, R3.3, and R7 using the larger aug-cc-pVQZ basis set (see Methods). We have confirmed at this level of theory that no barriers exist in the PESs of reactions R3.1 and R7 (Figures S9 and S10, respectively). The PES of reaction R3.2 in Figure 6 shows that a similar mechanism to R3.1 operates when the nitrate core ion is involved, followed by transfer of the HNO_3_ to the IO_3_^–^ end of the adduct over a submerged barrier. The geometries and molecular parameters of the species involved in the PESs of R3.1, R3.2, and R7 are provided in the Supporting Information.

It is also plausible that HNO_3_ adds to iodate core ions to form (HNO_3_)_*n*_·IO_3_^–^ with an increasing number of HNO_3_ ligands
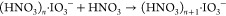
R8

In fact, the experiments in Figure S2 show that reducing the residence time in the ion–molecule reaction region enhances NO_3_^–^ relative to (HNO_3_)_*n*_·NO_3_^–^ (*n* = 1, 2), while the (HNO_3_)_*n*_·IO_3_^–^ (*n* = 1, 2) ions increase, which suggest that R8 is also a source of (HNO_3_)_*n*_·IO_3_^–^ (*n* = 2, 3) in our system, besides R3.2 and R3.3.

From the discussion above, it follows that masses 175 (IO_3_^–^), 238 (HNO_3_·IO_3_^–^), and 364 (I_2_O_3_·NO_3_^–^) may be sampling the same parent molecule. Figure S5 shows that these signals change in the same manner when the ozone concentration is doubled, which suggests that they indeed have common parent neutral molecules. Also, HNO_3_·IO_3_^–^ and I_2_O_3_·NO_3_^–^ are higher relative to IO_3_^–^ when the pressure is increased in the ion–molecule reaction volume, which is a result of enhanced ion–molecule clustering. Figure S4 indicates that the ratio of the IO_3_^–^ signal to the I_2_O_3_·NO_3_^–^ signal remains constant when changing the residence time of the gas in the I_*x*_O_*y*_ flow tube. These observations rule out the identification of mass 175 as a product of a reaction of I_*x*_O_*y*_ with water deposited on the reactor walls (i.e., HIO_3_).

The peaks at *m*/*z* = 222 and *m*/*z* = 285, which are minor in our experiments, were interpreted in previous CIMS work as resulting from ion–molecule reactions between HIO_2_ and (HNO_3_)_*n*_·NO_3_^–^ (*n* = 0–1) in the instrument inlet and as a proof of the presence of HIO_2_ in the sampled air. However, the *m*/*z* = 285 peak (HNO_3_·IO_2_^–^) may also originate from
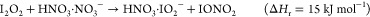
R6.3
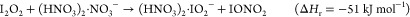
R6.4
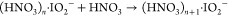
R9

Reaction R6.3 is essentially thermoneutral at the B3LYP/aug-cc-pVQZ level, with an accuracy of ±20 kJ mol^–1^. Higher level calculations are needed to determine whether this reaction is actually exothermic or not.

An important observation is the presence in the mass spectra of peaks at *m*/*z* = 251, *m*/*z* = 267, and *m*/*z* = 283, which have also been observed previously by nitrate CIMS,^4,11,23^ although no interpretation was given to them. These masses can be identified as the ion clusters IONO_2_·NO_3_^–^, OIONO_2_·NO_3_^–^, and O_2_IONO_2_·NO_3_^–^. We have seen that the *m*/*z* = 251 signal appears simply by adding I_2_ to the ion–molecule reaction zone, in line with the CIMS observations of Ganske et al.^34^ However, this signal also tracks the iodate core ion signals (Figure S5), which means that part of it is associated with the neutral chemistry in the I_*x*_O_*y*_ flow tube. In fact, iodine nitrate, IONO_2_, is a product of reaction R3, OIONO_2_ is a product of reaction R7, and O_2_IONO_2_ is a product of an analogous reaction of I_2_O_5_ and NO_3_^–^. Other nitrate core ion clusters of IONO_2_ and OIONO_2_ are also observed at *m*/*z* = 314 and *m*/*z* = 330, respectively. The interpretation of *m*/*z* = 251 as evidence of IONO_2_ not only brings closure to the proposed interpretation of the (HNO_3_)_*n*_·IO_3_^–^ CIMS signals in the dry experiments but also implies that it may be possible to use this signal to monitor IONO_2_ in the field.

The flow tube employed in this work is not suitable for studying the kinetics of I_*x*_O_*y*_ formation (to that end, the nitrate core ions should be in excess over iodine oxides). However, it can be seen that a longer residence time in the lower-pressure experiments enhances all the iodine-containing ions, indicating a general growth stage of the parent molecules (Figure S4). By contrast, in the higher-pressure, longer residence time experiments (Figure 3), the concentration of the parent higher-order oxides is higher relative to IO and OIO, which indicates higher concentrations of iodine oxides and faster second-order chemistry.

### Interpretation of Mass Spectra Obtained with Added Water Vapor: the Source of HIO_3_

Addition of water in the presence of light results in:

(a) the removal of ion signals associated with I_2_O_5_ (as previously observed by Sipilä et al.^23^)
(b) the increase in the iodate core ion signals (factor of ∼2 higher for the highest water concentration relative to the “dry” experiments) and
(c) the appearance of other ions that can be assigned to neutral I_*x*_O_*y*_. HIO_3_ adducts (also observed in previous nitrate CIMS studies,^4,11,23^ see Table 1)

These changes do not occur in the dark, where I_2_O_*y*_ (*y* = 2–4) but no I_2_O_5_ are formed. In addition, OIO and I_2_O_*y*_ (*y* = 2–4) are not removed by water. Hence, these observations suggest that I_2_O_5_ reacts with water to generate HIO_3_. The reaction between I_2_O_5_ and H_2_O is precluded by a large barrier in the PES,^30^ but recent ab initio calculations at the CCSD(T)//M06-2X/aug-ccpVTZ-PP + ECP28 level^32^ indicate that hydrolysis of I_2_O_5_ by the water dimer is feasible
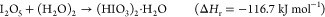
R10

Reaction R10 proceeds over a submerged barrier (−15.1 kJ mol^–1^). The complete process likely involves dissociation of the (HIO_3_)_2_·H_2_O complex, considering the exothermicity of reaction R10
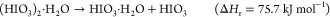
R11where we have used the bond energy of the HIO_3_·H_2_O complex^49^ computed at a similar level of theory than that used for reaction R10.

By contrast, our equivalent CCSD(T) calculations show that a second water molecule does not sufficiently reduce the height of the barrier of I_2_O_3_ + H_2_O PES (32 kJ mol^–1^ for one water molecule^22^ and 16 kJ mol^–1^ for the water dimer). This barrier is similar at lower levels of theory employed. Regarding I_2_O_4_ + (H_2_O)_2_, our B3LYP/6-311+G(2d,p) calculations indicate that a complex bound by 48 kJ mol^–1^ forms first and then rearranges over a submerged barrier (−44 kJ mol^–1^) to give
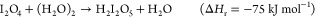
R12a
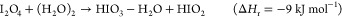
R12b

Dissociation of H_2_I_2_O_5_ to I_2_O_4_·H_2_O + H_2_O is endothermic by 89 kJ mol^–1^ and requires some rearrangement, so a barrier may be expected as well. This suggests that the peak at *m*/*z* = 398 corresponds in fact to H_2_I_2_O_5_·NO_3_^–^. The I_2_O_4_·H_2_O adduct formed directly from hydration of I_2_O_4_ is bound by 53 kJ mol^–1^^50^ and could also contribute to the signal at *m*/*z* = 398 in the high [H_2_O] experiments. Note however that the available I_2_O_4_ ion tracer (*m*/*z* = 443, I_2_O_4_·HNO_3_·NO_3_^–^) does not disappear by adding water (Figure 5c), which indicates that R12 is much slower than (R10 and R11).

The peak at *m*/*z* = 398 has also been interpreted as HIO_2_·HIO_3_·NO_3_^–^ and considered as evidence of the first HIO_2_–HIO_3_ neutral cluster.^4^ The proposed HIO_2_ ion tracers (*m*/*z* = 222 and *m*/*z* = 285) appear in the absence of water, suggesting that they are formed by R6 or other reactions involving I_*x*_O_*y*_. Their dependence on water is not completely consistent across different measurements. The signal at *m*/*z* = 285 (Figure S8c) generally increases when water is added. Reaction R12b would be a possible source of HIO_2_ in the presence of water. Hence, we cannot rule out that the peak at *m*/*z* = 398 is also representative of HIO_2_·HIO_3_·NO_3_^–^. We note nevertheless that the I_2_O_4_ concentration is expected to be significantly larger than that of HIO_2_, and hence it is more likely to contribute to clustering. Larger clusters with *m*/*z* > 500 amu (outside our instrumental range) reported in the CLOUD experiments^4^ can also be explained by addition of I_2_O_4_ to pre-existing clusters (see Table 2). It has been argued that the concentration of I_2_O_4_ in the CLOUD experiments was only 1% of that of HIO_3_ based on the comparison of anion signals. However, it is likely that the I_2_O_4_·NO_3_^–^ and I_2_O_4_·HNO_3_·NO_3_^–^ ion signals underestimate the I_2_O_4_ concentration and that part of I_2_O_4_ is actually observed as IO_3_^–^, as discussed above.

**Table 2:** Updated Mechanism of Iodine Gas-to-particle Conversion

chemistry	references and notes
I + O_3_ → IO + O_2_	evaluated kinetic and photochemical data for modeling of tropospheric iodine chemistry.^51^
IO + IO → I + OIO → I_2_O_2_	
IO + OIO ↔ I_2_O_3_	
OIO + OIO ↔ I_2_O_4_	
I_2_O_2_ + OIO → I_2_O_3_ + IO	the aggregation and dissociation rate constants of I_2_O_*y*_ + I_2_O_*z*_ reactions were calculated with the master equation solver MESMER using CCSD(T)//MP2/aug-cc-pVTZ energies, but the complete PES of these reactions was not explored.^50^ PIMS observations indicate that I_3_O_*y*_ (*y* = 4–7) molecules form rather than adducts with four iodine atoms.^18,22^ I_2_O_3_ was found to be very strongly bound and chemically stable to form weakly bound aggregates; hence, its fate remains unclear. The rate constants of some reactions involving I_2_O_*y*_ (*y* = 2–4) generating I_3_O_*y*_ (*y* = 4–7) were estimated by numerical modeling of I_*x*_O_*y*_ time traces obtained in flow tube experiments with PIMS detection.^22^ These semiquantitative estimates obtained from a tentative mechanism show that the rate constants of I_*x*_O_*y*_ aggregation reactions are close to the collision number. Analogous reactions of I_2_O_5_, not considered in previous work because this molecule was not detected, are now included in this table.
I_2_O_2_ + I_2_O_2_ → I_2_O_3_ + I_2_O	
I_2_O_4_ + OIO → I_3_O_6_	
I_2_O_4_ + I_2_O_4_ → I_3_O_6_ + OIO → I_3_O_7_ + IO	
I_2_O_4_ + I_2_O_5_ → I_3_O_7_ + OIO	
I_3_O_6_ + I_2_O_3_ ↔ I_5_O_9_	
I_3_O_6_ + I_2_O_4_ ↔ I_5_O_10_	
I_3_O_6_ + I_2_O_5_ ↔ I_5_O_11_	
I_3_O_7_ + I_2_O_3_ ↔ I_5_O_10_	
I_3_O_7_ + I_2_O_4_ ↔ I_5_O_11_	
I_3_O_7_ + I_2_O_5_ ↔ I_5_O_12_	
I_3_O_7_ + I_3_O_7_ → I_5_O_12_ + OIO	
I_2_O_4_ + (H_2_O)_2_ → H_2_I_2_O_5_ + H_2_O	H_2_I_5_O_2_ has been observed in previous work using nitrate CIMS, and it is also observed in the present work.
→ HIO_3_–H_2_O + HIO_2_	possible source of HIO_2_.
I_2_O_5_ + (H_2_O)_2_ → HIO_3_ + HIO_3_·H_2_O	source of HIO_3._ The PES of this reaction has been reported.^32,49^
HIO_3_ + HIO_2_ ↔ H_2_I_2_O_5_	theoretical estimates of the forward and reverse rate constants of the HIO_3_ + HIO_3_ and of HIO_3_ + I_2_O_4_ aggregation reactions have been reported.^22^ The I_2_O_*y*_·HIO_3_ adducts have been observed in the CLOUD chamber experiments using nitrate CIMS. They are also observed in the present work (*m*/*z* < 500 amu).
HIO_3_ + HIO_3_ ↔ (HIO_3_)_2_	
HIO_3_ + OIO ↔ OIO·HIO_3_	
HIO_3_ + I_2_O_2_ ↔ I_2_O_2_·HIO_3_	
HIO_3_ + I_2_O_3_ ↔ I_2_O_3_·HIO_3_	
HIO_3_ + I_2_O_4_ ↔ I_2_O_4_·HIO_3_	
HIO_3_ + I_2_O_5_ ↔ I_2_O_5_·HIO_3_	
I_2_O_4_ + H_2_O·HIO_3_ ↔ I_2_O_4_·H_2_O·HIO_3_	the (I_2_O_4_)_*n*_·H_2_O·(HIO_3_)_*m*_ adducts have been observed in the CLOUD chamber experiments using nitrate CIMS^4^ as anions with *m*/*z* > 500 amu (outside the mass range in the present work). The nucleation mechanism proceeds by addition of HIO_3_ and I_2_O_4_ to pre-existing molecular clusters.
H_2_I_2_O_5_ + HIO_3_ ↔ H_2_I_2_O_5_·HIO_3_	
H_2_I_2_O_5_ + I_2_O_4_ ↔ H_2_I_2_O_5_·I_2_O_4_	
H_2_I_2_O_5_ + H_2_I_2_O_5_ ↔ (H_2_I_2_O_5_)_2_	
I_2_O_4_ + I_2_O_4_ ↔ (I_2_O_4_)_2_	
I_2_O_4_·HIO_3_+ I_2_O_4_ ↔ (I_2_O_4_)_2_·HIO_3_	
H_2_I_2_O_5_·I_2_O_4_ + HIO_3_ ↔ H_2_I_2_O_5_·I_2_O_4_·HIO_3_	
H_2_I_2_O_5_·HIO_3_ + I_2_O_4_ ↔ H_2_I_2_O_5_·I_2_O_4_·HIO_3_	
H_2_I_2_O_5_·HIO_3_ + HIO_3_ ↔ H_2_I_2_O_5_·(HIO_3_)_2_	

photochemistry	references and notes
I_2_ + *h*ν → I + O	evaluated kinetic and photochemical data for modeling of tropospheric iodine chemistry.^51^
HOI + *h*ν → I + OH	
IO + *h*ν → I + O	
OIO + *h*ν → I + O_2_	
I_2_O_2_ + *h*ν → IO + IO	absorption cross-sections have been determined from experimental data and quantum calculations.^48^ The photolysis products have not been determined.
I_2_O_3_ + *h*ν → IO + OIO	
I_2_O_4_ + *h*ν → OIO + OIO	
I_2_O_5_ + *h*ν → IO_3_ + OIO	absorption cross-sections of I_2_O_5_ have not been determined in our previous experiments with PIMS because I_2_O_5_ was not detected.
I_3_O_6_ + *h*ν → I_2_O_5_ + IO	absorption cross-sections have been determined from experimental data and quantum calculations.^48^ The photolysis products have not been determined. Our new results indicate that I_2_O_5_ is a major photoproduct of I_*x*_O_*y*_ with *x* ≥ 3.
I_3_O_7_ + *h*ν → I_2_O_5_ + OIO	
I_5_O_12_ + *h*ν → I_3_O_7_ + I_2_O_5_	

### Comparison to PIMS Laboratory Experiments

In our previous work using PIMS^22^ with the same I_*x*_O_*y*_ source, we did not detect either I_2_O_5_ or HIO_3_ in the gas phase, and we did not observe cations that could be attributed to I_*x*_O_*y*_·HIO_3_ adducts. We argued that if there was a competition between clustering reactions of iodine oxides forming higher-order I_*x*_O_*y*_ and a fast reaction between iodine or iodine oxides and water-forming HIO_3_, there would have been a dramatic reduction in the I_*x*_O_*y*_-containing ions and a population of oxoacid clusters would have emerged. However, we observed only a limited reduction in the I_*x*_O_*y*_ signals and no reaction products when water was added. Water changed the composition of the particles to HIO_3_.^22^

Our present nitrate CIMS experiments indicate that this competition likely occurs between I_2_O_5_–I_*x*_O_*y*_ clustering and slow hydrolysis by the water dimer.^32^ The concentration of I_*x*_O_*y*_ in our flow tube is high (∼10^10^ to 10^12^ cm^–3^) compared to atmospheric conditions (10^7^ to 10^9^ cm^–3^). For low water concentrations, I_2_O_5_ and HIO_3_ are mainly removed by clustering with I_*x*_O_*y*_, and low concentrations of HIO_3_ and I_2_O_*y*_·HIO_3_ clusters exist, which may be too low to be detectable by PIMS in experiments with the same time scale as in the present ones. By contrast, in environmental chamber studies under MBL conditions, it is likely that even a slow water reaction with I_2_O_5_ dominates over clustering with I_*x*_O_*y*_, such that I_2_O_*y*_·HIO_3_ and HIO_3_ molecular clusters drive particle formation.

An important observation of the PIMS experiments is that particle formation is more intense when water is not added. This implies that I_*x*_O_*y*_ clusters form particles faster than I_2_O_*y*_·HIO_3_ and HIO_3_ clusters. Since the rate of formation of HIO_3_ likely depends on [H_2_O]^2^ this may have important atmospheric consequences for IOP formation in different environments.

### Comparison to Bromide CIMS Environmental Chamber Measurements

The signals observed by nitrate CIMS at *m*/*z* = 175 (IO_3_^–^), *m*/*z* = 238 (HNO_3_·IO_3_^–^), and *m*/*z* = 301 ((HNO_3_)_2_·IO_3_^–^) appear for very low water concentrations where iodine oxides are formed but not HIO_3_. These masses are also generated in the dark when I_2_O_5_ (the most likely precursor of HIO_3_) is not made. Water vapor does not remove I_2_O_*y*_ (*y* = 2, 3, and 4), but it does remove I_2_O_5_. At the same time, atmospheric water concentrations result in an increase in the *m*/*z* = 175 and *m*/*z* = 238 signals by a factor of 2 compared to dry conditions. Hence, the IO_3_^–^ core anions observed by CIMS are likely both products of the reaction of NO_3_^–^ with I_2_O_*y*_ (*y* = 2, 3, and 4) and with HIO_3_ in the instrument inlet and can be interpreted as the sum of iodine oxides I_2_O_*y*_ (*y* = 2, 3, and 4) and HIO_3_ present in the sampled air. A similar argument may apply to the IO_3_^–^ signal observed with a bromide CIMS in the CLOUD experiments^28^ since reactions between bromide ions and I_2_O_*y*_ are also exothermic, for example^22^
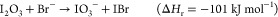
R13

In contrast, the HIO_3_·Br^–^ signal observed in the same experiments cannot result from
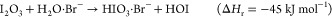
R14because this reaction is precluded by a barrier of 20 kJ mol^–1^, according to our quantum calculations at the B3LYP/aug-cc-pVQZ level. Hence the HIO_3_·Br^–^ anion appears to be a genuine HIO_3_ tracer.

### Atmospheric Implications

The IOP formation mechanism proposed in our previous work^22^ can now be updated by adding the source of I_2_O_5_ and HIO_3_ and the two molecular cluster formation pathways (Table 2). Further experimental and theoretical work is required to investigate the photolysis products of higher-order iodine oxides, the specific fate of I_2_O_2_ and I_2_O_3_, and the rate constants of the I_*x*_O_*y*_, HIO_3_, and I_*x*_O_*y*_·HIO_3_ clustering reactions.

CIMS observations should help in better constraining atmospheric iodine models since the most relevant species (IO, OIO, I_*x*_O_*y*_, and HIO_3_) can be detected with this technique with high sensitivity. Laboratory and chamber experiments using spectroscopic instrumentation should be conducted in order to calibrate the CIMS signals of these key species. Comparison between bromide and nitrate CIMS observations of iodate core ions may help in quantifying the fraction of the signal of these ions that can be attributed to I_2_O_*y*_ and HIO_3_ under different atmospherically relevant conditions. Our 26 Torr experiments, where almost all I_2_O_5_ is depleted when atmospherically relevant water concentrations are added, indicate that ∼50% of the IO_3_^–^ and HIO_3_^–^ signals observed by CIMS correspond to I_2_O_*y*_ (*y* = 2–4).

The observation of the signal at mass 251 in our experiments is also particularly relevant for the CIMS observations in the context of atmospheric chemistry. We have interpreted this signal as IONO_2_·NO_3_^–^, where IONO_2_ is a product of the reaction between I_2_O_3_ and NO_3_^–^. Formed in the atmosphere through the recombination of IO and NO_2_, IONO_2_ is also a key iodine reservoir and a carrier of iodine toward the aerosol phase in polluted and semi-polluted regions. To our knowledge, no measurements of this compound have been reported to date, and in fact, no in situ technique has been developed to detect it, in contrast to, for example, ClONO_2_.^52^ Baccarini et al.^11^ observed a strong signal at *m*/*z* = 251 using nitrate CIMS, which was attributed to the O_6_N_2_I^–^ anion but not explicitly to IONO_2_·NO_3_^–^. We propose that this was possibly the first measurement of IONO_2_ reported in the literature. Further experiments should determine the relative contribution to that signal of ambient IONO_2_ and IONO_2_ formed in the CIMS inlet from I_2_O_3_ + NO_3_^–^.

Our previous results using PIMS indicated that clustering of iodine oxides leads to particle formation. Water is not required to form nucleating molecules, which has implications for where in the atmosphere IOP formation can take place. Since IOP formation is not limited by water abundance, it can occur in the polar MBL, as observed,^11^ and perhaps also in the upper troposphere. Most other new particle formation processes (e.g., sulfuric acid, ammonia) depend directly or indirectly on the presence of water. A particle mechanism that does not depend on water may significantly contribute to, even dominate, total new particle formation in water-limited regions, even with small amounts of iodine. This may be the reason why iodine is the dominant nucleating species in the high Arctic.^11^ Note that, in addition, water-limited regions will generally be associated with lower pre-existing aerosol loadings, thereby increasing the survival chance of any newly formed iodine particle. A recent experimental study indicates that the transition between the dry and humid IOP formation mechanisms occurs at around 20% RH.^53^

## Conclusions

Our flow tube experiments reveal that the iodate core ion signals measured by nitrate CIMS are contributed both by I_2_O_*y*_ and HIO_3_ neutral molecules. They also indicate a plausible photolytic and water-dependent source of HIO_3_, which is consistent with the coexistence of iodine oxides and oxoacids in nitrate CIMS spectra obtained under MBL conditions, as well as with PIMS laboratory observations with typically higher iodine oxide concentrations. In addition, they show that the formation of HIO_3_ under high water and low iodine concentrations leads to the formation of I_2_O_*y*_·HIO_3_ clusters, which are the likely precursors of iodine particles in the MBL. Under dry conditions, I_*x*_O_*y*_ clusters lead to different, faster nucleation. These results fill the gaps in the mechanism that connects inorganic and organic iodine emissions and IOPs, which greatly facilitates the implementation of iodine chemistry and iodine-driven nucleation in atmospheric models. This should eventually enable the radiative forcing of IOPs to be computed for the first time.

## Supporting Information Available

Additional experimental details, PESs of selected reactions and geometries, and molecular properties and heats of formation of relevant stationary points (PDF)
