# Supplementary material for: Insights into the Chemistry of Iodine New Particle Formation: The Role of Iodine Oxides and the Source of Iodic Acid
Source: J Am Chem Soc. 2022 May 23;144(21):9240–53. doi: 10.1021/jacs.1c12957 (PMC9164234; doi:10.1021/jacs.1c12957)
Supplement: Supplementary file 1 — ja1c12957_si_001.pdf [file ja1c12957_si_001.pdf]

# Supplementary Materials for

## Insights into the chemistry of iodine new particle formation: the role of iodine oxides and the source of iodic acid.

Juan Carlos Gómez Martín*, Thomas R. Lewis[2], Alexander D. James, Alfonso Saiz-Lopez* and John M. C. Plane

*Corresponding authors. Email: jcgomez@iaa.es, a.saiz@csic.es

**This PDF file includes:**

Figs. S1 to S10
Tables S1 to S3

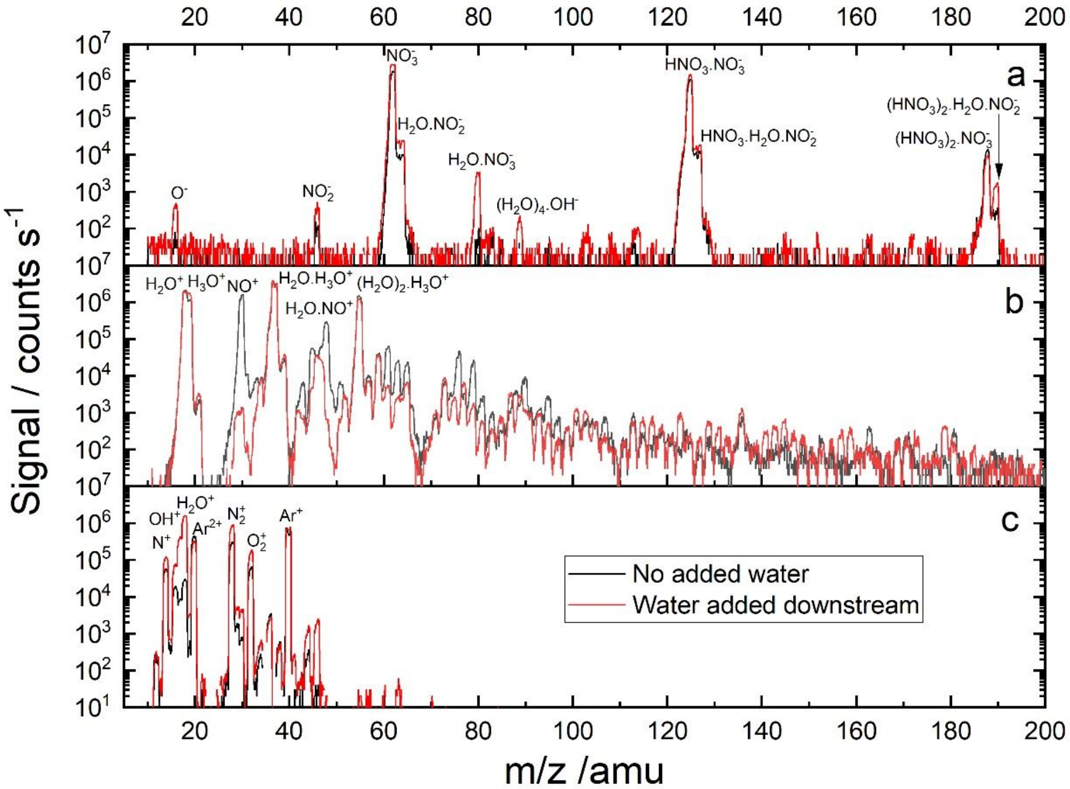


**Fig. S1.** Characterization of the nitrate core anion source response to water vapor. Panel a: negative ions. Panel b: positive ions. Panel c: residual gas analysis (RGA), using electron impact ionization. Black curves: no added water. Red curves: water added 15 cm downstream of the nitric acid injection. Concentration in the ion-molecule reactive region of the order of $10^{15}$ molecule $cm^{-3}$.

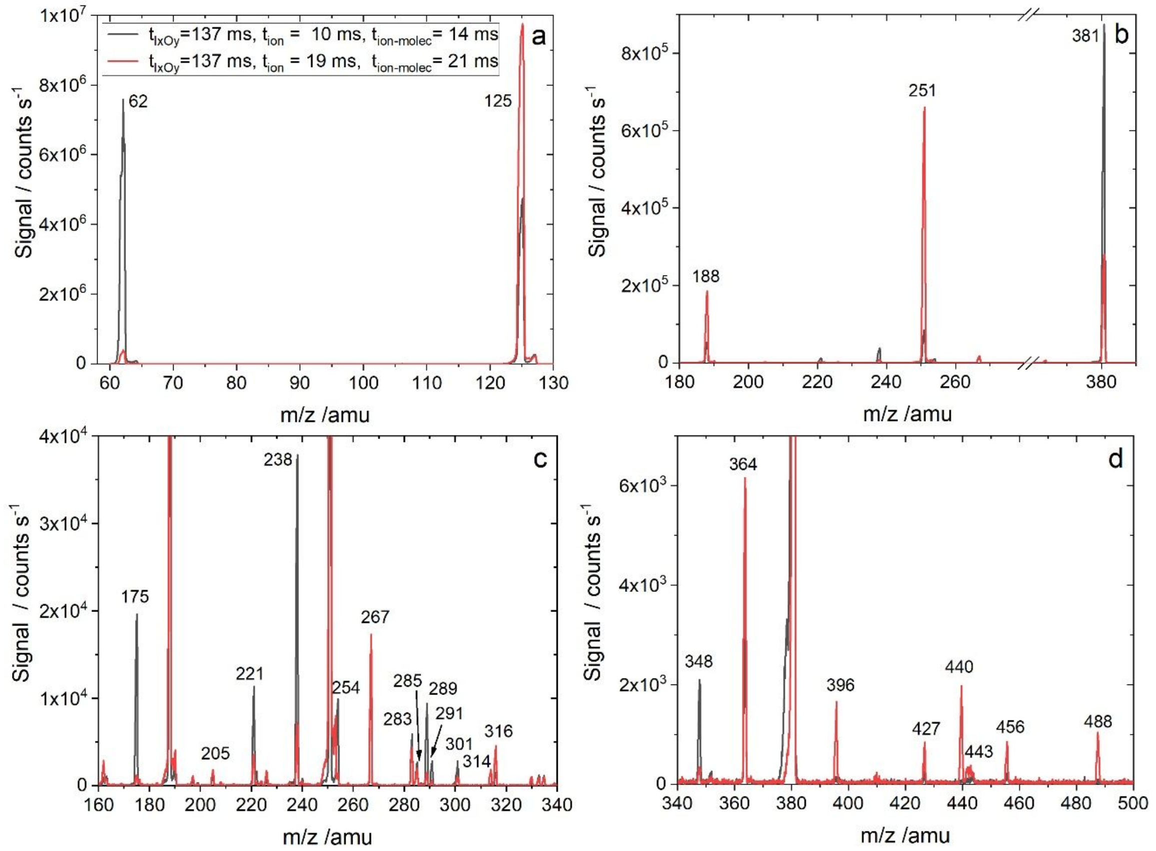


**Fig. S2.** Dependence of ion signal on residence time in the ion-molecule reaction region of the flow tube. Each panel focus on a different set of peaks: main nitrate core ions (panel a), nitrate anion + molecular iodine reaction products ($IONO_2.NO_3^-$ and $I_3^-$), main iodine-containing anions including iodate core ions (panel c) and iodine oxide-nitrate core ion clusters (panel d). Black lines: 14 ms residence time. The main masses are indicated by numbers. Red lines: 21 ms residence time. Iodine oxides are produced under the same conditions in both experiments.

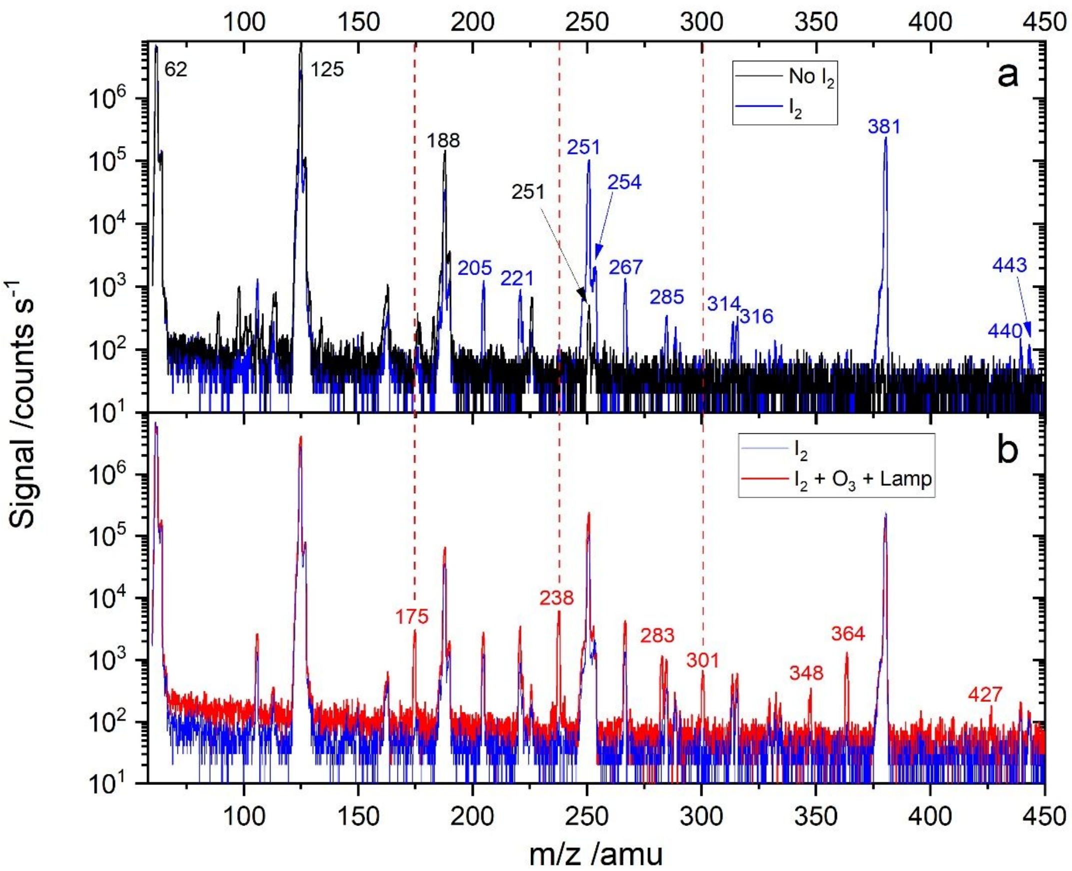


**Fig. S3.** Characterization of the nitrate core anion source response to molecular iodne and iodine oxides. Panel a: clean source mass spectrum (black line) and effect of molecular iodine (blue line). Panel b: source plus molecular iodine mass spectrum (blue line) and full chemistry mass spectrum with iodine oxides (red line).

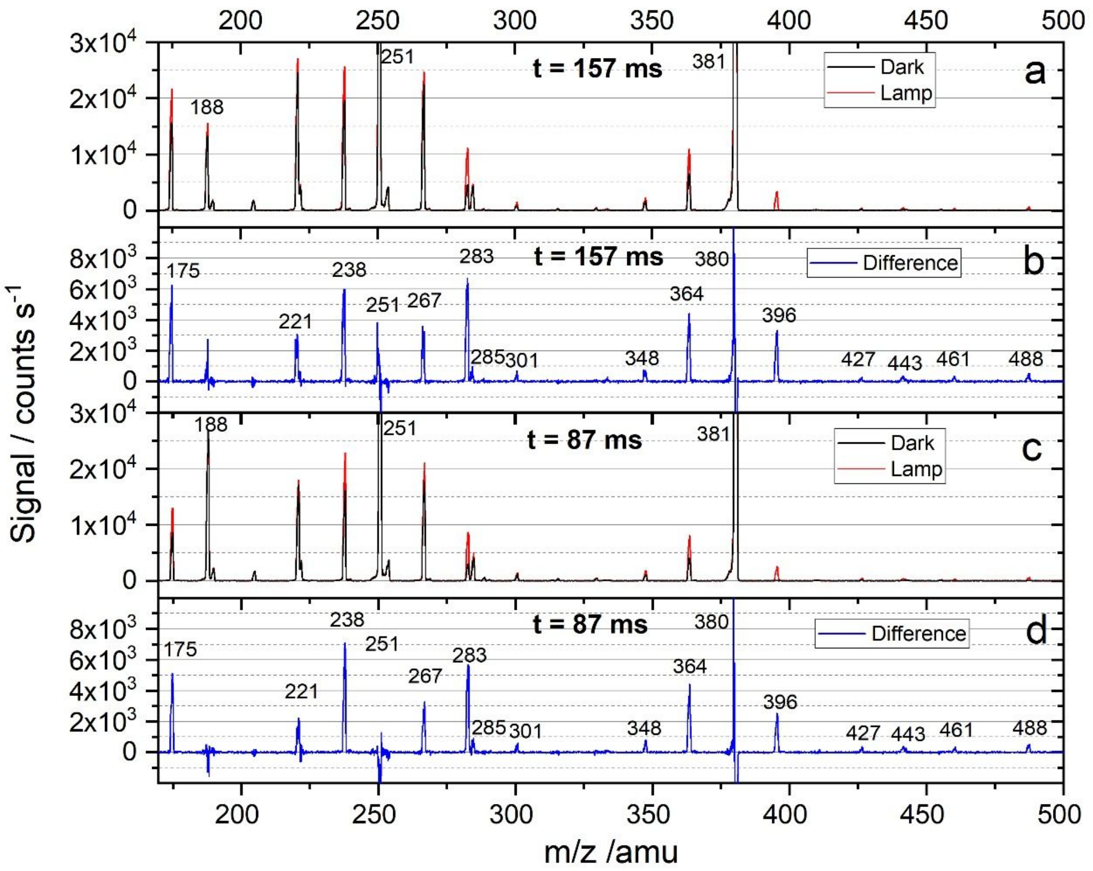


**Fig. S4.** Dependence of ion signal on residence time in the $I_xO_y$ flow tube at 3 Torr. Panels a and b: 157 ms residence time. Panels c and d: 87 ms residence time. Panels a and c show the raw mass spectra with and without light (red and black lines, respectively). Panels b and d show the difference spectra between the spectra with and without light, i.e. the photolytic signal. The residence time in the ion-molecule reaction region is the same in both experiments.

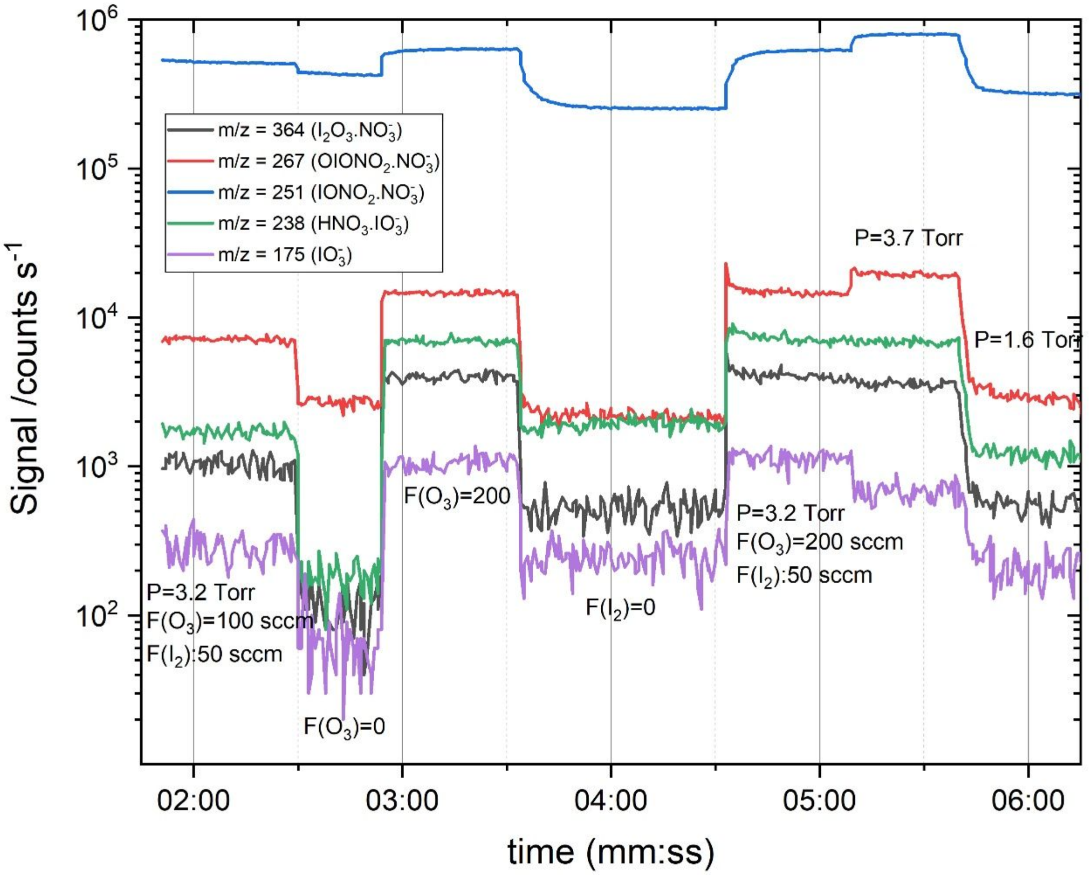


**Fig. S5.** Time traces of the peak signals of selected ions under different $I_xO_y$ formation conditions, as indicated in the plot. Flows (F) and pressure (P) are varied to investigate the effect on the selected ions.

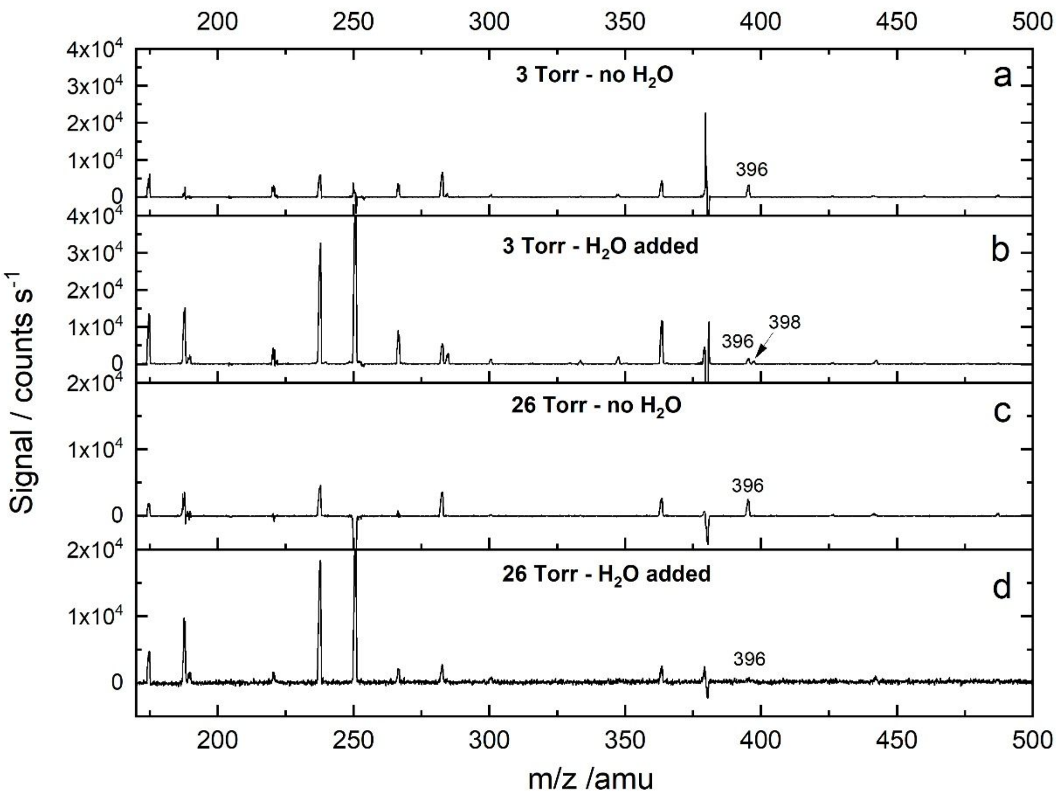


**Fig. S6.** Photolytic signals with and without added water. Panels a and b: 3 Torr experiments with and without added water, respectively. Panels c and d: the same for 26 Torr experiments.

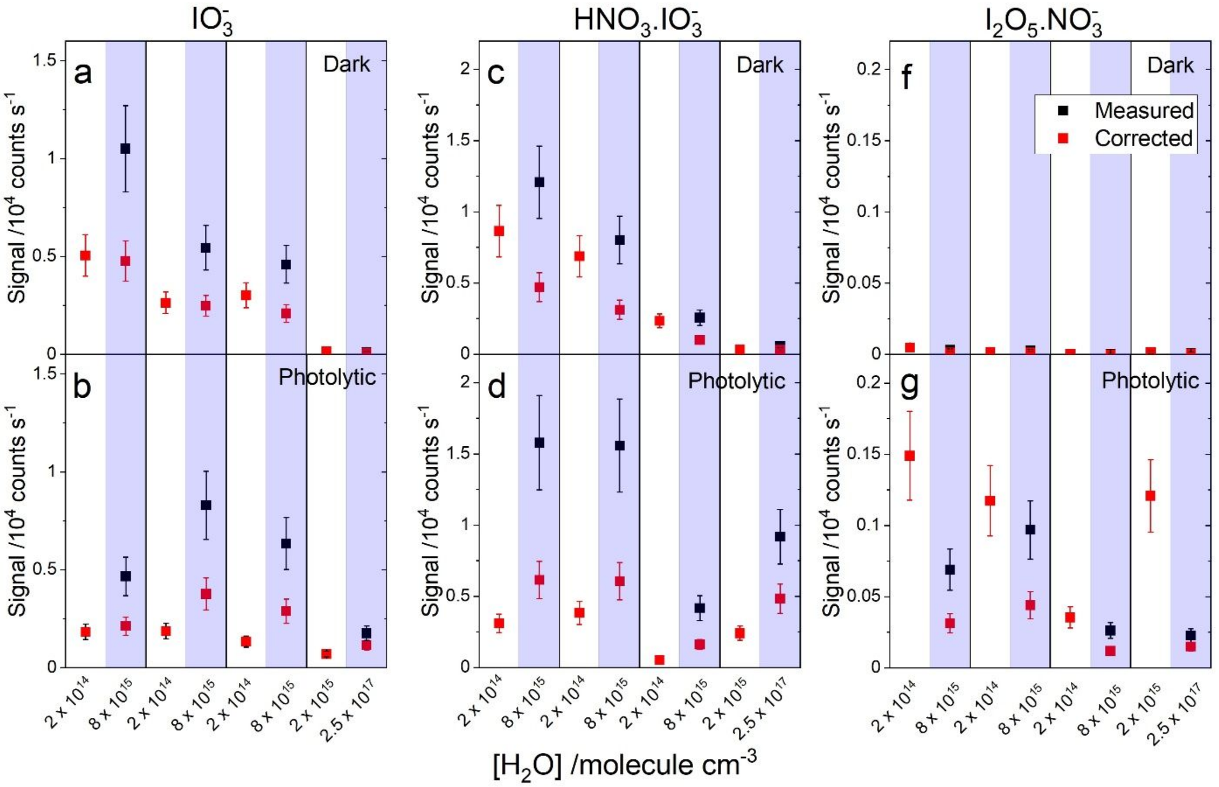


**Fig. S7.** Water dependence of dark (panels a, c, d) and photolytic signals (panels b, d, c) for m/z = 175 ($IO_3^-$, panels a and b), m/z = 238 ($HNO_3.IO_3^-$, panels c and d) and m/z = 396 ($I_2O_5.NO_3^-$, panels f and g) for three experiments at 3 Torr ([$H_2O$] = 2 × $10^{14}$ molecule $cm^{-3}$ and [$H_2O$] = 8 × $10^{15}$ molecule $cm^{-3}$) and one experiment at 26 Torr ([$H_2O$] = 2 × $10^{15}$ molecule $cm^{-3}$ and [$H_2O$] = 2.5 × $10^{17}$ molecule $cm^{-3}$). Black squares: signals obtained by integrating the corresponding mass peaks. Red squares: signals corrected with the $NO_3^-$ ratios with/without water shown in Fig. 4 of the main text. Blue shaded regions indicate experiments with water added to the carrier flow.

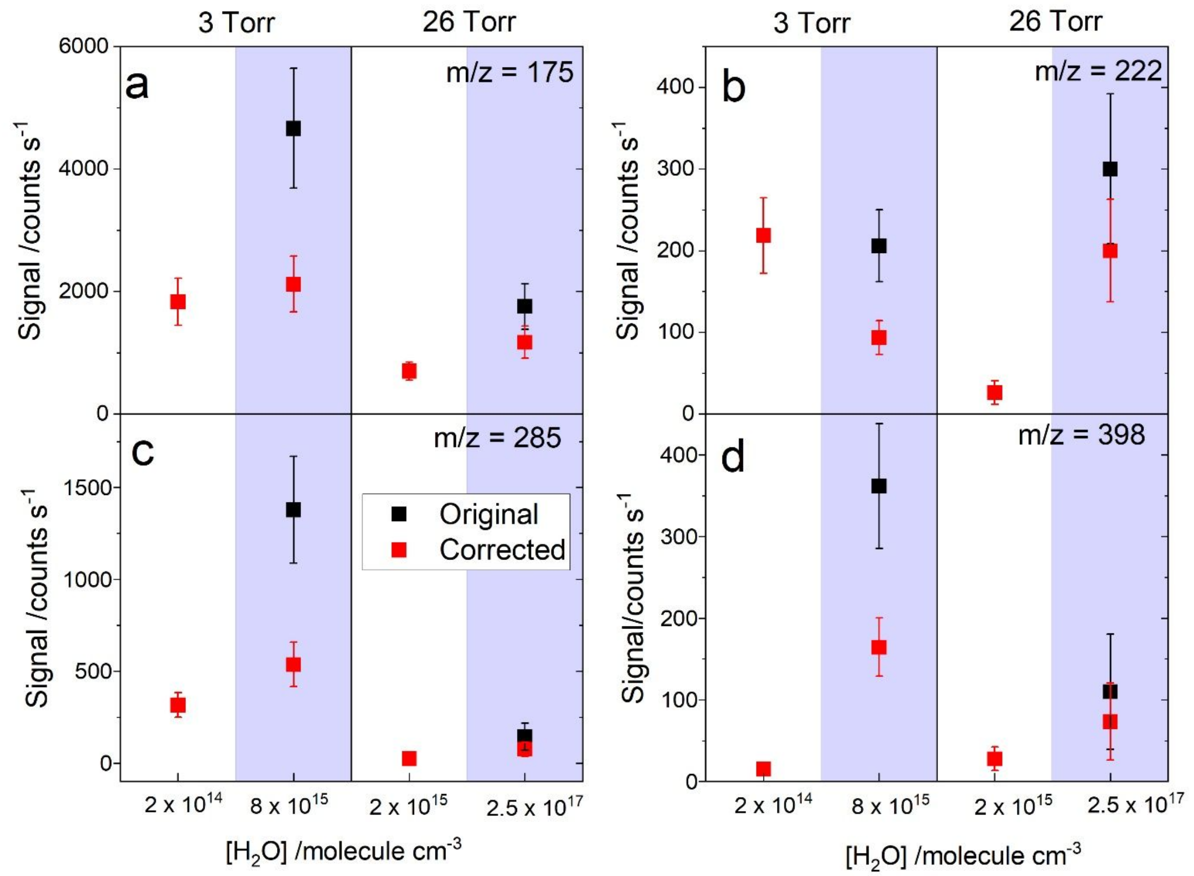


**Fig. S8.** Water dependence of photolytic signals for m/z = 175 ($IO_3^-$, panel a), m/z = 222 ($HNO_3.IO_2^-$ or $HIO_2.NO_3^-$, panel b), m/z = 285 ($(HNO_3)_2.IO_2^-$ or $HIO_2.HNO_3.NO_3^-$, panel c) and m/z = 398 ($I_2O_4.H_2O.NO_3^-$ or $HIO_2.HIO_3.NO_3^-$, panel d) for two experiments at 3 Torr and 26 Torr. Black squares: signals obtained by integrating the corresponding mass peaks. Red squares: signals corrected with the $NO_3^-$ ratios with/without water shown in Fig. 4 of the main text.

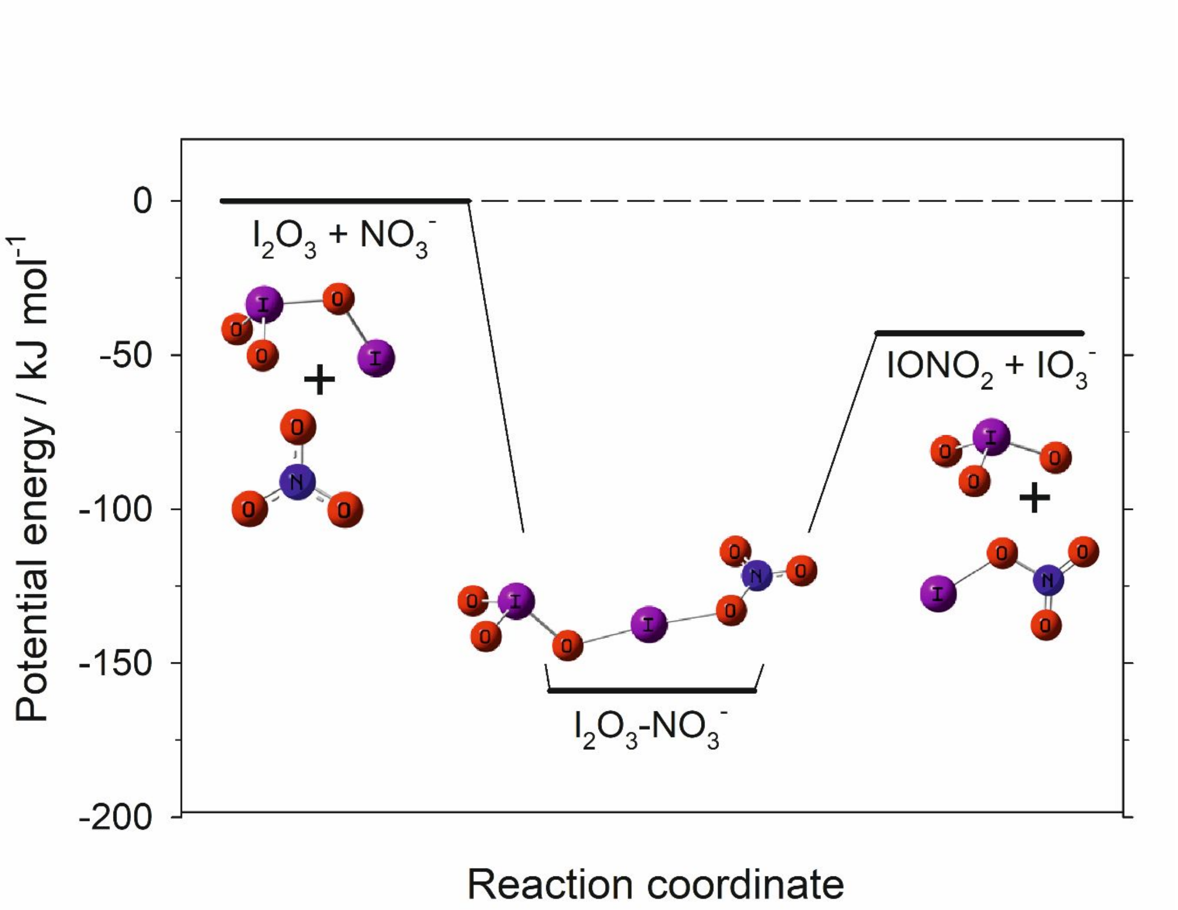


**Figure S9.** Potential energy for the reaction $I_2O_3 + NO_3^- \rightarrow IONO_2 + IO_3^-$ (see Table S1 for further details).

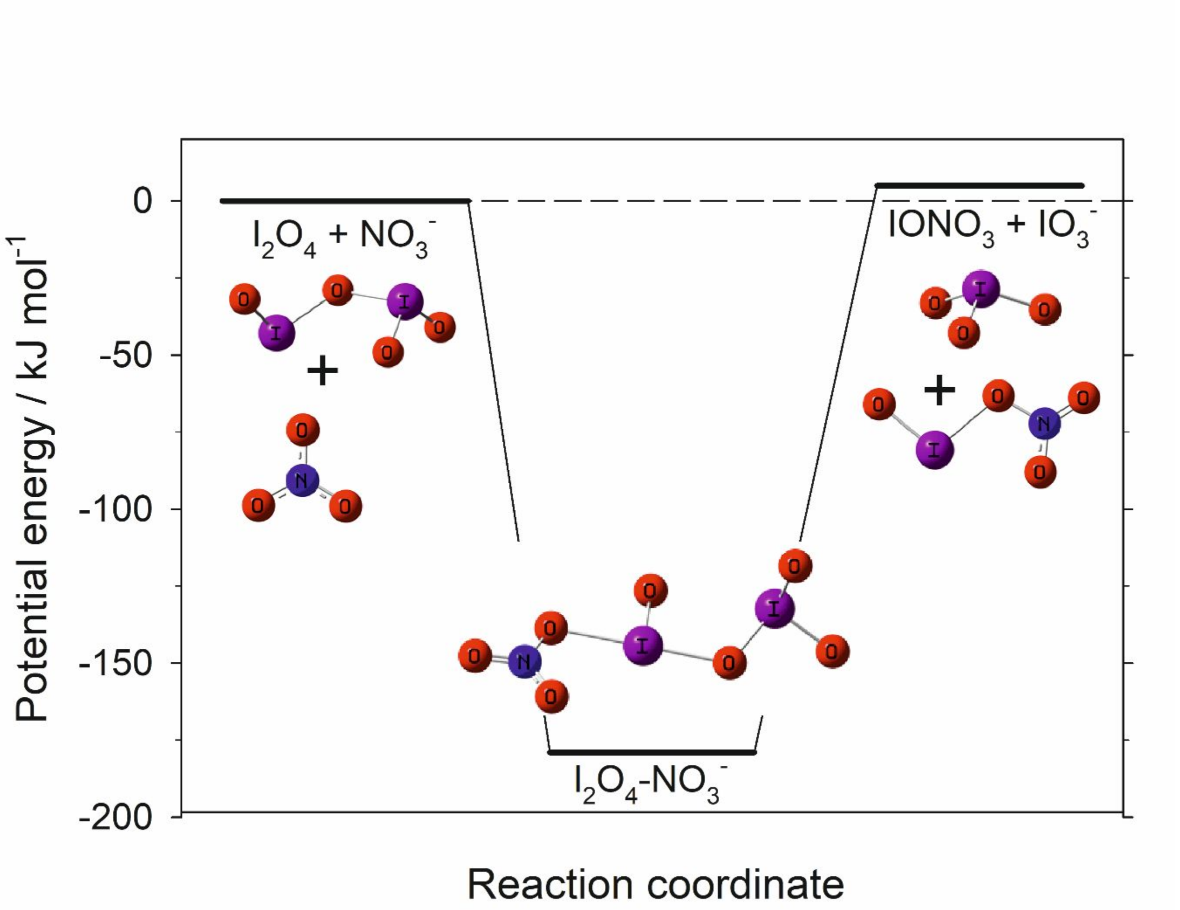


**Figure S10.** Potential energy for the reaction $I_2O_4 + NO_3^- \rightarrow IONO_3 + IO_3^-$ (see Table S2 for further details).

**Table S1.** Molecular properties and heats of formation (at 0 K) of the stationary points on the $I_2O_3 + NO_3^- \rightarrow IONO_2 + IO_3^-$ potential energy surface (singlet spin multiplicity). The geometries are illustrated in Figure S1.

| Molecule (electronic state) | Geometry (Cartesian co-ordinates in Å) [a] | Rotational constants (GHz) [a] | Vibrational frequencies ($cm^{-1}$) [a] | Relative energy (kJ $mol^{-1}$, inc. zero point energy) |
|---|---|---|---|---|
| $I_2O_3$ ($^1A'$) | I, 0.181, -1.535, 0.<br>O, 1.040, 0.246, 0.<br>I, -0.077, 1.921, 0.<br>O, -0.865, -1.452, 1.446<br>O, -0.865, -1.452, -1.446 | 4.47465<br>0.57273<br>0.55951 | 34, 85, 237, 253, 297, 441, 637, 878, 907 | 0 |
| $NO_3^-$ ($^1A_1'$) | N, 0., 0., 0.<br>O, 1.091, -0.630, 0.<br>O, 0., 1.260, 0.<br>O, -1.091, -0.630, 0. | 13.2647<br>13.2647<br>6.63237 | 703, 703, 842, 1062, 1373, 1373 | - |
| $I_2O_3.NO_3^-$ complex | I, 2.180, -0.188, -0.300<br>O, 0.918, -0.009, 1.096<br>I, -1.257, -0.292, 0.636<br>O, 3.275, 1.212, -0.009<br>O, 3.094, -1.675,0.149<br>N, -4.213, 0.149, -0.305<br>O, -3.434, -0.733, 0.324<br>O, -5.388, -0.183, -0.428<br>O, -3.730, 1.199, -0.710 | 2.62598<br>0.21670<br>0.21077 | 21, 39, 51, 72, 95, 152, 164, 197, 248, 259, 302, 410, 668, 705, 726, 804, 846, 869, 976, 1297, 1548 | -159 [a] |
| $IONO_2$ ($^1A'$) | N, 0.002, 0., 0.0<br>O, 0.002, 0., 1.199<br>O, 0.902, 0., -0.789<br>O, -1.270, 0., -0.684<br>I, -2.942, 0., 0.460 | 12.3620<br>1.30507<br>1.18045 | 113, 177, 365, 588, 727, 752, 826, 1310, 1702 | -43 [b] |
| $IO_3^-$ ($^1A_1$) | I, 0., 0., -0.220<br>O, 1.456, -0.841, 0.481<br>O, -1.456, -0.841, 0.481<br>O, 0., 1.682, 0.481 | 5.9437<br>5.9437<br>3.7217 | 278, 278, 321, 795, 817, 817 | - |

[a] Calculated at the B3LYP/6-311+g(2d,p) level of theory [1], using the G2 all electron basis set for I [2].

[b] Calculated at the B3LYP/aug-cc-pVQZ level of theory [1], using the aug-cc-pVQZ basis set for I from Peterson et al. [3].

**Table S2.** Molecular properties and heats of formation (at 0 K) of the stationary points on the $I_2O_4 + NO_3^- \rightarrow IONO_3 + IO_3^-$ potential energy surface (singlet spin multiplicity). The geometries are illustrated in Figure S2.

| Molecule (electronic state) | Geometry (Cartesian co-ordinates in Å) [a] | Rotational constants (GHz) [a] | Vibrational frequencies ($cm^{-1}$) [a] | Relative energy (kJ $mol^{-1}$, inc. zero point energy) |
|---|---|---|---|---|
| $I_2O_4$ ($^1A$) | O 1.077 1.140 1.213<br>I 1.658 0.184 -0.184<br>O -0.092 -0.566 -0.802<br>O 2.451 -1.300 0.417<br>I -1.824 -0.233 0.175<br>O -2.514 1.247 -0.645 | 3.60865<br>0.48681<br>0.46973 | 34, 67, 93, 161, 241, 258, 306, 437, 528, 810, 867, 901 | 0 |
| $NO_3^-$ ($^1A_1'$) | N, 0., 0., 0.<br>O, 1.091, -0.630, 0.<br>O, 0., 1.260, 0.<br>O, -1.091, -0.630, 0. | 13.2647<br>13.2647<br>6.63237 | 703, 703, 842, 1062, 1373, 1373 | - |
| $I_2O_4.NO_3^-$ complex | O, -2.720, -0.411, 1.696<br>I, -2.402, -0.169, -0.059<br>O, -0.904, 1.030, -0.043<br>O, -3.660, 0.968, -0.670<br>I, 1.068, 0.149, -0.055<br>O, 0.334, -1.477, -0.554<br>N, 4.110, 0.051, 0.129<br>O, 5.259, -0.364, 0.092<br>O, 3.789, 1.217, 0.398<br>O, 3.147, -0.812, -0.138 | 2.89697<br>0.21489<br>0.21040 | 26, 31, 52, 66, 72, 97, 155, 167, 189, 220, 271, 273, 304, 440, 631, 705, 730, 746, 809, 848, 870, 1007, 1297, 1533 | -179 [a] |
| $IONO_3$ ($^1A'$) | N, 0.481, -1.975, 0.0<br>O, -0.625, -1.157, 0.0<br>O, 0.253, -3.150, 0.0<br>O, 1.569, -1.403, 0.0<br>O,-1.621, 1.706, 0.0<br>I, 0.003, 0.874, 0.0 | 7.62862<br>1.16563<br>1.01113 | 59, 116, 133, 177, 321, 669, 733, 773, 818, 912, 1276, 1638 | 4.9 [b] |
| $IO_3^-$ ($^1A_1$) | I, 0., 0., -0.220<br>O, 1.456, -0.841, 0.481<br>O, -1.456, -0.841, 0.481<br>O, 0., 1.682, 0.481 | 5.9437<br>5.9437<br>3.7217 | 278, 278, 321, 795, 817, 817 | - |

[a] Calculated at the B3LYP/6-311+g(2d,p) level of theory [1], using the G2 all electron basis set for I [2].

[b] Calculated at the B3LYP/aug-cc-pVQZ level of theory [1], using the aug-cc-pVQZ basis set for I from Peterson et al. [3].

**Table S3.** Molecular properties and heats of formation (at 0 K) of the stationary points on the $I_2O_3 + NO_3^-.HNO_3 \rightarrow IONO_2 + IO_3^-.HNO_3$ potential energy surface (singlet spin multiplicity). The geometries are illustrated in Figure S3.

| Molecule (electronic state) | Geometry (Cartesian co-ordinates in Å) [a] | Rotational constants (GHz) [a] | Vibrational frequencies ($cm^{-1}$) [a] | Relative energy (kJ $mol^{-1}$, inc. zero point energy) |
|---|---|---|---|---|
| $I_2O_3$ ($^1A'$) | I, 0.181, -1.535, 0.<br>O, 1.040, 0.246, 0.<br>I, -0.077, 1.921, 0.<br>O, -0.865, -1.452, 1.446<br>O, -0.865, -1.452, -1.446 | 4.47465<br>0.57273<br>0.55951 | 34, 85, 237, 253, 297, 441, 637, 878, 907 | 0 |
| $NO_3^-.HNO_3$ | N, -2.014, 0.0590, 0.0338<br>O, -1.110, -0.497, -0.730<br>O, -3.160, -0.394, -0.019<br>O, -1.683, 1.001, 0.761<br>H, 0.070, 0.025, -0.612<br>N, 2.013, -0.067, 0.0396<br>O, 1.1055, 0.497, -0.744<br>O, 1.674, -0.999, 0.762<br>O, 3.148, 0.392, -0.025 | 6.21000<br>0.84546<br>0.83345 | 31, 50, 52, 129, 156, 230, 558, 695, 702, 731, 817, 826, 861, 1030, 1072, 1357, 1391, 1453, 1503, 1550, 1685 | - |
| $I_2O_3$-$NO_3^-.HNO_3$ complex | N, -7.207, -0.343, 0.453<br>O, -6.132, -1.059, 0.896<br>O, -8.284, -0.795, 0.776<br>O, -6.989, 0.655, -0.204<br>H, -5.293, -0.601, 0.554<br>N, -3.262, -0.328, -0.770<br>O, -3.800, -0.023, 0.319<br>O, -2.076, 0.131, -1.030<br>O, -3.797, -1.043, -1.605<br>I, 1.786, 3.624, 1.680<br>O, 0.103, 2.775, 1.895<br>I, -0.951, 1.495, 0.479<br>O, 2.961, 2.290, 1.399<br>O, 1.652, 4.555, 0.146 | 1.19172<br>0.09483<br>0.09127 | 16, 18, 26, 31, 39, 48, 63, 66, 102, 108, 145, 154, 180, 239, 260, 266, 292, 393, 644, 662, 690, 717, 735, 799, 809, 852, 876, 969, 985, 1055, 1281, 1345, 1434, 1514, 1687, 2937 | -92 [a] |
| TS | N, -2.280, 2.800, -0.395<br>O, -2.587, 1.813, 0.508<br>O, -2.948, 3.799, -0.286<br>O, -1.401, 2.571, -1.203<br>H, -1.922, 1.101, 0.366 | 0.49319<br>0.22185<br>0.16299 | -70*i*, 23, 35, 41, 45, 49, 56, 73, 79, 104, 117, 155, | -69 [a] |

| | | | | |
|---|---|---|---|---|
| | N, -3.135, -2.429, 0.417<br>O, -3.389, -1.235, 0.532<br>O, -1.898, -2.807, 0.085<br>O, -3.936, -3.341, 0.583<br>I, 1.692, 1.786, -0.008<br>O, 1.323, 0.131, -0.840<br>I, -0.345, -1.241, -0.288<br>O, 0.425, 1.894, 1.272<br>O, 3.269, 1.499, 0.815 | | 172, 190,<br>250, 274,<br>315, 392,<br>492, 614,<br>666, 684,<br>707, 727,<br>793, 803,<br>846, 871,<br>958, 977,<br>1294, 1341,<br>1363, 1550,<br>1693, 3537 | |
| $IONO_2$-$IO_3^-$<br>.$HNO_3$ complex | N, -1.553, 4.995, -0.849<br>O, 2.505, 4.188, -0.344<br>O, -1.925, 6.088, -1.215<br>O, -0.404, 4.565, -0.898<br>H, -2.044, 3.280, -0.064<br>N, -1.685, -5.080, 0.598<br>O, -1.008, -5.141, -0.416<br>O, -1.842, -3.895, 1.220<br>O, -2.261, -6.016, 1.136<br>I, 0.202, 1.526, 0.194<br>O, 0.103, -0.203, -0.476<br>I, -0.887, -2.108, 0.397<br>O, -1.562, 1.983, 0.376<br>O, 0.808, 1.368, 1.876 | 1.18402<br>0.11775<br>0.11165 | 11, 20, 29,<br>37, 46, 55,<br>60, 73, 79,<br>143, 163,<br>174, 193,<br>238, 275,<br>280, 321,<br>404, 658,<br>697, 702,<br>721, 740,<br>777, 796,<br>806, 873,<br>954, 1001,<br>1057, 1297,<br>1317, 1495,<br>1570, 1694,<br>2276 | -112 [a] |
| $IONO_2$<br>($^1A'$) | N, 0.002, 0., 0.0<br>O, 0.002, 0., 1.199<br>O, 0.902, 0., -0.789<br>O, -1.270, 0., -0.684<br>I, -2.942, 0., 0.460 | 12.3620<br>1.30507<br>1.18045 | 113, 177,<br>365, 588,<br>727, 752,<br>826, 1310,<br>1702 | -15 [b] |
| $IO_3^-$.$HNO_3$ | I, 1.022, 0.147, 0.153<br>O, 2.570, -0.647, 0.620<br>O, 1.330, 0.995, -1.403<br>O, 0.085, -1.429, -0.417<br>H, -0.924, -1.245, -0.457<br>N, -2.761, 0.080, 0.073<br>O, -2.474, -1.028, -0.496<br>O, -1.815, 0.793, 0.511<br>O, -3.936, 0.431, 0.187 | 3.56764<br>0.61422<br>0.58766 | 34<br>58<br>67<br>93<br>157<br>195<br>262<br>298<br>327<br>650<br>710 | - |

| | | | | |
|---|---|---|---|---|
| | | | 731<br>832<br>852<br>872<br>950<br>1063<br>1289<br>1382<br>1481<br>2702 | |

[a] Calculated at the B3LYP/6-311+g(2d,p) level of theory [1], using the G2 all electron basis set for I [2].

[b] Calculated at the B3LYP/aug-cc-pVQZ level of theory [1], using the aug-cc-pVQZ basis set for I from Peterson et al. [3].

## References


(1) Frisch, M. J.; Trucks, G. W.; Schlegel, H. B.; Scuseria, G. E.; Robb, M. A.; Cheeseman, J. R.; Scalmani, G.; Barone, V.; Petersson, G. A.; Nakatsuji, H.; Li, X.; Caricato, M.; Marenich, A. V.; Bloino, J.; Janesko, B. G.; Gomperts, R.; Mennucci, B.; Hratch, D. J. Gaussian 16 Rev. C.01. *Wallingford CT* **2016**.

(2) Glukhovtsev, M. N.; Pross, A.; McGrath, M. P.; Radom, L. Extension of Gaussian-2 (G2) Theory to Bromine- and Iodine-Containing Molecules: Use of Effective Core Potentials. *J. Chem. Phys.* **1995**, *103* (5), 1878.

(3) Peterson, K. a.; Shepler, B. C.; Figgen, D.; Stoll, H. On the Spectroscopic and Thermochemical Properties of ClO, BrO, IO, and Their Anions. *J. Phys. Chem. A* **2006**, *110* (51), 13877–13883. https://doi.org/10.1021/jp065887l.
